# LncRNA *EPR* regulates intestinal mucus production and protects against inflammation and tumorigenesis

**DOI:** 10.1093/nar/gkad257

**Published:** 2023-04-18

**Authors:** Paola Briata, Luca Mastracci, Ettore Zapparoli, Luca Caputo, Elisa Ferracci, Alessandra Silvestri, Anna Garuti, Meriem Hadjer Hamadou, Alberto Inga, Elisa Marcaccini, Federica Grillo, Gabriele Bucci, Pier Lorenzo Puri, Galina Beznoussenko, Alexander Mironov, Fulvio Chiacchiera, Roberto Gherzi

**Affiliations:** Gene Expression Regulation Laboratory, IRCCS Ospedale Policlinico San Martino, 16132 Genova, Italy; Pathology Unit, IRCCS Ospedale Policlinico San Martino, 16132 Genova, Italy; Pathology Unit, Department of Surgical and Diagnostic Sciences (DISC), University of Genoa, Genova, Italy; Center for Omics Sciences, IRCCS Ospedale San Raffaele, 20132 Milano, Italy; Development, Aging and Regeneration Program, Sanford Burnham Prebys Medical Discovery Institute, La Jolla, CA 92037, USA; Laboratory of Stem Cells and Cancer Genomics, Department of Cellular, Computational and Integrative Biology (CIBIO), University of Trento, Trento, Italy; IRCCS Humanitas Research Hospital, Via Manzoni 56, 20089 Rozzano, Milano, Italy; Translational Genomics, IRCCS Ospedale Policlinico San Martino, 16132 Genova, Italy; Department of Cellular, Computational and Integrative Biology (CIBIO), University of Trento, Trento, Italy; Department of Cellular, Computational and Integrative Biology (CIBIO), University of Trento, Trento, Italy; Gene Expression Regulation Laboratory, IRCCS Ospedale Policlinico San Martino, 16132 Genova, Italy; Pathology Unit, IRCCS Ospedale Policlinico San Martino, 16132 Genova, Italy; Pathology Unit, Department of Surgical and Diagnostic Sciences (DISC), University of Genoa, Genova, Italy; Center for Omics Sciences, IRCCS Ospedale San Raffaele, 20132 Milano, Italy; Development, Aging and Regeneration Program, Sanford Burnham Prebys Medical Discovery Institute, La Jolla, CA 92037, USA; The AIRC Institute of Molecular Oncology, Via Adamello 16, 20139 Milano, Italy; The AIRC Institute of Molecular Oncology, Via Adamello 16, 20139 Milano, Italy; Laboratory of Stem Cells and Cancer Genomics, Department of Cellular, Computational and Integrative Biology (CIBIO), University of Trento, Trento, Italy; Gene Expression Regulation Laboratory, IRCCS Ospedale Policlinico San Martino, 16132 Genova, Italy

## Abstract

The long non-coding RNA *EPR* is expressed in epithelial tissues, binds to chromatin and controls distinct biological activities in mouse mammary gland cells. Because of its high expression in the intestine, in this study we have generated a colon-specific conditional targeted deletion (*EPR* cKO) to evaluate *EPR in vivo* functions in mice. *EPR* cKO mice display epithelium hyperproliferation, impaired mucus production and secretion, as well as inflammatory infiltration in the proximal portion of the large intestine. RNA sequencing analysis reveals a rearrangement of the colon crypt transcriptome with strong reduction of goblet cell-specific factors including those involved in the synthesis, assembly, transport and control of mucus proteins. Further, colon mucosa integrity and permeability are impaired in *EPR* cKO mice, and this results in higher susceptibility to dextran sodium sulfate (DSS)-induced colitis and tumor formation. Human *EPR* is down-regulated in human cancer cell lines as well as in human cancers, and overexpression of *EPR* in a colon cancer cell line results in enhanced expression of pro-apoptotic genes. Mechanistically, we show that *EPR* directly interacts with select genes involved in mucus metabolism whose expression is reduced in *EPR* cKO mice and that *EPR* deletion causes tridimensional chromatin organization changes.

## INTRODUCTION

The development of high-throughput sequencing analyses has led to the identification of thousands of transcriptionally active genomic regions that are not protein-coding genes yet produce non-coding RNAs (1). Among them, long non-coding RNAs (lncRNAs), arbitrarily defined by a length of at least 500 nucleotides, share structural features with mRNAs, and their expression often changes in individual tissues and cell types, and at distinct differentiation stages (2). LncRNAs regulate several biological functions—as diverse as cell proliferation, cell cycling and apoptosis—by controlling chromatin remodeling, transcriptional and post-transcriptional processes, as well as protein function (3,4). Altered lncRNA expression has been related to the dysregulation of crucial cellular circuitries and may be involved in multiple human diseases including cancer (5,6). Thousands of lncRNA loci have been identified in the mammalian genome, but investigating their modes of action has been a challenge. Recent studies highlighted the potential role of lncRNAs in normal intestine and colorectal cancer, and their function as molecular links between inflammation and colorectal carcinogenesis has been hypothesized (7–9).

We have recently described a previously uncharacterized mammalian lncRNA expressed in epithelial tissues (BC030870, ENSMUSG00000074300) that we termed *EPR* (after Epithelial Program Regulator) (10). *EPR* interacts with the RNA-binding protein KHSRP, counteracts transforming growth factor-β (TGF-β)-induced epithelial to mesenchymal transition in mammary gland cells and contains an open reading frame (ORF) that is translated into a small peptide localized at epithelial cell junctions (10). We found that *EPR* regulates the expression of a large set of target transcripts independently of peptide biogenesis (10). Our studies have revealed that *EPR* interacts with chromatin, regulates *Cdkn1a* gene expression by affecting both its transcription and mRNA decay, and controls cell proliferation in both immortalized and transformed mammary gland cells as well as tumor growth in a mouse model of orthotopic transplantation (10). More recently, by integrating data derived from chromatin isolation by RNA purification (ChIRP)-Seq, ChIP-Seq as well as RNA-Seq in a comprehensive analysis, we identified a group of bona fide direct transcriptional *EPR* targets in mammary gland cells and studied, among them, *Mettl7a1*, defining its role in translation (11,12).

*EPR* expression is prominent in the gastro-enteric tract and, to obtain insight into *EPR* function in an animal model, we set out to abrogate *EPR* expression in the mouse large intestine. To this end, we generated a colon epithelium-specific conditional *EPR* knockout (*EPR* cKO) mouse line. *EPR* cKO mice display epithelial hyperproliferation accompanied by inflammatory infiltration and impaired mucus production in the proximal portion of the colon. We also show that *EPR* conditional deletion leads to a strong reduction of goblet cell-specific factors including those involved in the synthesis, assembly, transport and control of mucus. Further, the colon mucosa integrity and permeability are impaired in *EPR* cKO mice which are highly susceptible to pharmacologically induced colitis and tumor formation. Some of the genes whose expression is transcriptionally reduced in *EPR* cKO mice are directly bound by lncRNA, and *EPR* deletion causes a rearrangement of the tridimensional (3D) chromatin organization.

## MATERIALS AND METHODS

### *EPR* conditional knockout in the large intestine

The *EPR* locus was modified using homologous recombination in murine embryonic stem (ES) cells. Blastocyst injection of targeted ES cells yielded chimeric founder mice, which were bred to C57BL/6 wild-type mice (this part of the experiment was performed at Polygene Transgenetics, Switzerland). Heterozygous mice were crossed to mice hemizygous for the CDX2P-NLS Cre transgene (13) (catalogue # 009350 The Jackson Laboratory). The CDX2P-NLS Cre transgene contains 9.5 kb promoter/enhancer sequence of the human CDX2 gene directing the expression of CRE recombinase (13). After two additional rounds of breeding, we obtained *EPR* fl/fl (used as control throughout this study) and *EPR* fl/fl;CDX2P-NLS Cre (the colon-specific conditional *EPR* knockout that we named *EPR* cKO) mice. Genotyping was routinely performed using a polymerase chain reaction (PCR)-based strategy.

All mice were maintained under pathogen-free conditions and all experiments involving animals were performed in accordance with the Organismo Preposto al Benessere degli Animali (OPBA) of IRCCS Ospedale Policlinico San Martino. Experimental protocols were approved by national regulators (Authorization # 716/2019-PR).

### Animal treatments

#### Dextran sodium sulfate (DSS)-induced colitis model

Twelve-week-old mice (both males and females) were treated with different concentrations (ranging from 1.5% to 2.5%) of DSS (40 kDa; Sigma-Aldrich) (14) dissolved in drinking water for different intervals of time up to 7 days. Mice were then euthanized for histological analysis.

#### Azoxymethane (AOM)/DSS-induced mouse colorectal tumorigenesis model

Twelve-week-old mice (both males and females) were given a single intraperitoneal injection of AOM (10 mg/kg body weight, dissolved in saline; Sigma-Aldrich) (15,16). Starting 1 week after AOM injection, mice were treated with 1.5% DSS in drinking water for 4 d and were euthanized 14 weeks after the end of the DSS treatment. Dissected large intestines were fixed in 10% formalin and embedded in paraffin for histopathological analysis. Body weight, the presence of occult or gross blood per rectum, stool consistency and mortality were monitored daily during treatments to evaluate the necessity of an ethical endpoint of experiments.

### Histological and immunohistochemical analysis

Intestine was dissected immediately after death, explanted, flushed with ice-cold phosphate-buffered saline (PBS), fixed in buffered 10% formaldehyde overnight at 4°C, routinely processed and paraffin embedded. For histological staining, 5 μm thick sections were rehydrated and stained with hematoxylin and eosin (H&E) or Alcian Blue and periodic acid–Shiff's (AB PAS). For immunohistochemical analysis, heat-induced antigen unmasking was performed using Tris-EDTA buffer (10 mM Tris Base, 1 mM EDTA solution, 0.05% Tween 20, pH 9.0). Sections were incubated overnight with primary antibodies, washed and subsequently incubated with horseradish peroxidase polymer-conjugated secondary antibodies. Nuclei were counterstained using hematoxylin, and images were acquired using bright field microscopy (Nikon). Cell counts were performed by randomly acquiring non-overlapping images and by counting the number of positive cells for each field using the imaging analysis software package ImageJ 1.53a (http://imagej.nih.gov/ij, NIH).

### Colonic crypt preparation

Crypts were isolated from 12- to 16-week-old mice according to the protocol described by Mahe and co-workers (17).

### Cell lines, plasmids and transfections

Colon adenocarcinoma cell lines SW480 (ICLC HTL99017), HCT116 (ICLC HTL95025), HCT-15 (ICLC HTL00001), CACO-2 (ICLC HTL97023) and HT-29 (ICLC HTL99026) were obtained from the Interlab Cell Line Collection at the IRCCS Ospedale Policlinico San Martino (Genova, Italy). SW480 cells stably overexpressing either full-length *EPR* [from nucleotide 1 to 1487 of murine BC030870 (10), named SW480-*EPR*] or *EPR*STOPM [*EPR* mutated as described in (10), named SW480-*EPR*STOPM] as well as cells stably transfected with the empty vector (SW480-empty vector) were maintained in selective medium containing 1000 μg/ml G418 (Sigma-Aldrich). Transfections were performed using Lipofectamine 3000 (ThermoFisher).

### Antibodies and preparation of cell extracts

Antibodies used in this study are listed in Supplementary Table S1. Cell extracts were prepared from purified colon crypts using lysis buffer [50 mM Tris–HCl, pH 8.0, 0.5% Triton X-100, 150 mM NaCl supplemented with complete protease and phosphatase inhibitors (Roche)].

### Clonogenic assay

The clonogenic (or colony-forming) assay was performed essentially as summarized below. Cells were plated in 6-well multi-well plates (in sextuplicate). The number of cells plated has been established based on pilot experiments conducted in empty vector-transfected cells to obtain from 15 to 100 colonies per well after at least six replications. Colonies were stained with 2 ml 0.01% (w/v) crystal violet in H_2_O for 30 min and counted using the imaging analysis software package ImageJ 1.53a (http://imagej.nih.gov/ij, NIH).

### Transmission electron microscopy (TEM)

Electron microscopy analysis of samples was performed exactly as described previously (18). Briefly, samples were immediately placed into the fixative composed of 2.5% formaldehyde and 2.5% glutaraldehyde in 0.1 M sodium cacodylate buffer (pH 7.4) (Electron Microscopy Sciences) for at least 24 h. Samples were post-fixed in a 4% paraformaldehyde and 2.5% glutaraldehyde (Electron Microscopy Sciences) mixture in 0.2 M sodium cacodylate pH 7.2 for 2 h, followed by six washes in 0.2 M sodium cacodylate pH 7.2 at room temperature. Then samples were incubated in a 1:1 mixture of 2% osmium tetroxide and 3% potassium ferrocyanide for 1 h followed by rinsing six times in 0.2 M cacodylate buffer at room temperature. Next, samples were sequentially treated with 0.3% thiocarbohydrazide in 0.2 M cacodylate buffer for 10 min and 1% OsO_4_ in 0.2 M cacodylate buffer (pH 6.9) for 30 min, rinsed with 0.1 M sodium cacodylate (pH 6.9) buffer until the complete removal of all traces of the yellow osmium fixative, washed in deionized water, treated with 1% uranyl acetate in water for 1 h and washed in water again (19). Samples were subsequently subjected to dehydration in ethanol and acetone, and embedded in epoxy resin at room temperature, and the resin was polymerized for at least 72 h in a 60°C oven. Samples were then sectioned with a diamond knife (Diatome) using an ultramicrotome (Leica). Ribbons of serial sections were transferred to Formvar-coated, 1 × 2 mm slot grids. Grids were imaged in a Tecnai-F20 transmission electron microscope. The number of Golgi cisternae was counted in 20 distinct sections per each biological replicate, and the statistical significance of differences among *EPR* fl/fl and *EPR* cKO mice was calculated using Student's *t*-test.

### Immunofluorescence and confocal microscopy

Intestinal samples were fixed overnight in paraformaldehyde, l-lysine pH 7.4 and NaIO_4_ (PLP buffer). They were then washed, dehydrated in 20% sucrose for at least 4 h and embedded in OCT compound (Sakura). Cryosections of 10 μm were rehydrated, blocked with 0.1 M Tris–HCl pH 7.4, 2% fetal bovine serum and 0.3% Triton X-100, and stained with specific antibodies. Primary antibodies were incubated overnight at 4°C and slices were then incubated with the appropriate fluorophore-conjugated secondary antibody. Before imaging, nuclei were counterstained with 4′,6-diamidino-2-phenylindole (DAPI) and slides were mounted in VECTASHIELD^®^ Mounting Media (Vector Laboratories). Coverslips were permanently sealed around the perimeter with nail polish. Slides were stored at 4°C in the dark till acquisition by laser scanning confocal microscopy performed on a Leica TCS SP8 equipped with 405, 488, 552 and 638 nm diode lasers. Images were acquired with an HC PL FLUOTAR ×40/1.30 oil immersion objective. The Fiji software package was used for image analysis.

### FITC–dextran permeability assay

Mice were starved in the morning for 4 h and then ∼400 mg/kg fluorescein isothiocyanate (FITC)–dextran (4 kDa; Sigma-Aldrich) was orally administered through gavage. Blood was collected from the tail vain after 4 h and the concentration of FITC–dextran in plasma samples was measured as fluorescence intensity (CLARIOstar Plus Microplate Reader; BMG Labtech).

### Total RNA preparation and quantitative reverse transcription–PCR (qRT–PCR) analysis

Total RNA was extracted from isolated mouse colon crypts, total mouse large intestine, ileum and cultured cell lines using the TriPure reagent (Roche). Reverse transcription was carried out on 100 ng of total RNA using Transcriptor Reverse Transcriptase (Roche) and random hexamers in a total volume of 10 μl according to the manufacturer's instructions. Quantitative PCR was performed using the Luna Universal qPCR master mix (NEB), and the Realplex II Mastercycler (Eppendorf) according to the manufacturer's instructions in a total volume of 10 μl. Forty cycles were performed with denaturation (95°C for 15 s) and extension (60°C for 30 s). A melting curve was conducted for each assay (60–95°C ramp). The sequence-specific primers utilized for PCR are listed in Supplementary Table S2 and were synthesized by TIB MolBiol. In some preliminary experiments, the colon was dissected into four distinct sections of equal length along the proximal–distal axis. Total RNA was isolated from each section and analyzed by qRT–PCR.

Total RNA was extracted from 10 adenocarcinoma samples (and the corresponding samples of the surrounding normal tissue) that were randomly and anonymously selected from the Genoa Tissue Bank at the Ospedale Policlinico San Martino. RNA was reverse transcribed as above and analyzed by qPCR using the specific primers listed in Supplementary Table S2. To analyze the expression of human *EPR*, we used four distinct pairs of primers spanning different regions of the lncRNA (Supplemetary Table S2).

### RNA isolation from cytoplasm, nucleoplasm and chromatin

We followed the protocol published by Corey and co-workers (20) to extract RNA from isolated large intestine crypts. Both cytoplasmic and nucleoplasmic RNAs were precipitated and washed with ice-cold 70% (v/v) ethanol prior to being dissolved in TriPure reagent (Roche), while the chromatin pellets were immediately dissolved in TriPure. A 10 μl aliquot of 0.5 M EDTA was added to all the samples in TriPure and heated to 65°C with vortexing until dissolved (∼10 min).

### RNA deep-sequencing (RNA-Seq) and RNA-Seq analysis

Colon was isolated from either six *EPR* fl/fl or six *EPR* cKO 12-week-old mice and flushed with ice-cold PBS. The proximal third of each colon was excised and immediately frozen in dry ice. Then, the organ fragments were homogenized using a motorized pestle (Sigma-Aldrich) in the presence of TriPure reagent, and high-quality RNA was extracted. A total of 12 libraries were prepared using the standard Illumina Stranded Total RNA Prep with Ribo-Zero Plus protocol, and sequenced on an Illumina NovaSeq 6000. Image analysis and base calling were performed using the Illumina NovaSeq Control Software. This approach yielded between 30 and 70 million reads that were further processed. Raw reads were trimmed at the ends to remove low-quality calls with Trimnomatic v0.39. Paired-end reads were mapped with STAR v2.5.3a to the indexed mm10 genome. R v3.5.1 and Bioconductor v3.8 were used for secondary analysis. The featureCounts function from the Rsubread v1.6.4 package was used to assign read counts to the genes of the Ensembl GRCm38.97 gene annotation. Only transcripts with at least 1 cpm (counts per million) in at least four samples were considered. We kept *EPR* fl/fl versus *EPR* cKO differentially expressed transcripts when the observed Bayesian statistic was significant [Benjamini and Hochberg-corrected *P*-value <0.001; |logfold change (FC)| > 1.25].

In the case of cultured colon cancer cells, high-quality RNA was extracted from either empty vector or *EPR*-overexpressing SW480 cells (biological triplicates for each experimental condition), and a total of six libraries were prepared using the standard Illumina Stranded Total RNA Prep with Ribo-Zero Plus protocol, and sequenced on an Illumina NovaSeq 6000. Subsequent processing and analyses were performed as described above. We kept SW480-empty vector versus SW480-*EPR* differentially expressed transcripts when the observed Bayesian statistic was significant (Benjamini and Hochberg-corrected *P*-value <0.001; |logFC| > 1.25).

### Cleavage under targets and release using nuclease (CUT&RUN)

CUT&RUN experiments were performed in two biological replicates using the Cell Signaling Technology CUT&RUN Kit following the manufacturer's instructions. Briefly, crypts from either *EPR* fl/fl or *EPR* cKO mice proximal colon were isolated and 5 mg of lightly fixed tissue (0.1% formaldehyde for 2 min at room temperature) were used for each experimental point. Upon incubation with Concanavalin A-coated beads and with freshly dissolved digitonin, cells were incubated (16 h at 4°C under rotation) with either anti-H3K27ac (Ab4729 from Abcam), anti-H3K27me3 (#9733 Cell Signaling Technology) or negative control rabbit (DA1E) monoclonal antibody (mAb) IgG XP^®^ Isotype Control (Cell Signaling Technology). pAG-MNase enzyme was added and activated to digest targeted regions of the genome. DNA was purified from input and enriched chromatin samples using the GFX PCR DNA and gel band Purification kit (Cytiva), and quantified prior to being utilized in qPCRs using the primers listed in Supplementary Table S2.

### Chromatin isolation by RNA purification (ChIRP) and high-throughput sequencing

ChIRP was performed by optimizing the protocol published in Zapparoli *et al.* (11) to large intestine crypts. Briefly, crypts were isolated from the proximal third of the colon of either *EPR* fl/fl or *EPR* cKO mice, washed four times with ice-cold PBS and cross-linked in 1% glutaraldehyde in PBS at room temperature for 10 min on an end-to-end rotator. After glutaraldehyde quenching and repeated PBS washes, crypt pellets were weighed and resuspended in 1.0 ml of complete Lysis Buffer [50 mM Tris–HCl pH 7.0, 10 mM EDTA, 1% SDS, 1× Complete (Roche), 500 U of RNase inhibitor] per each 100 mg of cell pellet. Cell suspensions were sonicated for 90 min (power set to 70%) and the sonicated cell lysate was centrifuged at 16 100 *g* at 4°C for 10 min. Lysates were divided into two 1 ml aliquots, transferred into polypropylene tubes, mixed with 2 ml of Complete Hybridization Buffer (750 mM NaCl, 1% SDS, 50 mM Tris–HCl, pH 7.0, 1 mM EDTA, 15% formamide, 1× Complete, 1000 U of RNase inhibitor) and hybridized with 1 μl (100 pmol) of either EVEN or ODD pools of 20-mer 3′ Bio-TEG DNA oligonucleotides designed with the single-molecule FISH online designer (Stellaris) (11), respectively. Hybridization was carried out at 37°C for 4 h under continuous shaking. Since the two pools of ODD and EVEN probes are different, the two respective pools of chromatin pulled down by off-target hybridization would be different, while only the chromatin DNA associated with EPR would be commonly pulled down by both the ODD and EVEN probes (11). A 70 μl alquot of pre-washed C-1 magnetic beads (Thermo Fisher) was added to each hybridization mixture for 30 min at 37°C under continuous shaking. Beads were immobilized and washed four times for 5 min at 37°C with shaking [wash buffer: 2× NaCl and sodium citrate (SSC), 0.5% SDS, 1× Complete]. While one aliquot (10% of the material) was utilized for RNA extraction, the remaining 90% was subject to DNA purification by incubating each bead pellet twice with 150 μl of Complete DNA Elution Buffer (50 mM NaHCO_3_, 1% SDS, 25 μg/ml RNase A, 100 U/ml RNase H) for 30 min at 37°C with shaking. Eluted DNA was incubated with Proteinase K (1 mg/ml final dilution) for 45 min at 50°C with shaking, extracted with phenol/chloroform/isoamylalchool and ethanol precipitated. ChIRP was performed in triplicate, and precipitated DNA was subjected to qPCR analysis using specific primers (Supplementary Table S2).

### Promoter capture-HiC

Hi-C was performed in crypts from mouse proximal colon using the Arima-HiC Kit according to the manufacturer's instructions (Arima Genomics). Briefly, crypts were isolated from two distinct *EPR* fl/fl and two *EPR* cKO mice, snap-frozen and weighed (100–130 μg/sample). A 100 μg aliquot of crypts was resuspended in PBS and cross-linked in 2% formaldehyde for 10 min at room temperature, digested with a restriction enzyme cocktail, end-labeled with Biotin-14-dATP and then ligated. The ligated chromatin was reverse-cross-linked and fragmented using Bioruptor (Diagenode) to obtain an average fragment size of 400 bp. Fragmented DNA was then size-selected to have a size distribution between 200 and 600 bp, and finally subjected to biotin enrichment. Then, DNA libraries were prepared according to the procedure detailed in the Arima Capture-HiC Kit User Guide for mammalian cells (https://arimagenomics.com/wp-content/files/User-Guide-Arima-Capture-HiC-for-Mammalian-Cell-Lines.pdf). The Arima Mouse Promoter Panel was designed to the promoters of 25 752 genes from the Mouse GRCm38 Ensemble database, version 94, including: 21 088 protein-coding genes, 207 antisense RNAs, 544 long intergenic non-coding RNAs (lincRNAs), 1015 microRNAs (miRNAs), 1494 small nucleolar RNAs (snoRNAs) and 1383 small nuclear RNAs (snRNAs). Capture probes were designed to the restriction fragment of each of the promoters. The probes were manufactured using 1x tiling with repeat masking and balance boosting. Arima Capture-HiC libraries were sequenced via Illumina sequencers in ‘paired-end’ mode and sequence analyzed according to the Arima Capture-HiC Analysis Pipeline. Data were pre-processed using HiCUP (21) and loops called using CHiCAGO (22) at Arima Genomics.

### 3C-PCR

3C-PCR experiments were conducted as described in Hagege *et al.* (23). The sequence of the primers used is presented in Supplementary Table S2.

### Statistical analysis

All the graphs, calculations and statistical analyses were performed using GraphPad Prism software version 9.0 for MacOS (GraphPad Software).

### Analysis of single-cell RNA-Seq data in mouse and human as well as of protein expression in human colon adenocarcinoma

Information about single-cell RNA-Seq analysis in mouse large intestine was obtained through the web-based access to the *Tabula Muris* Consortium data [https://tabula-muris.ds.czbiohub.org (24)]. The expression of human *EPR* (LINC01207 aka SMIM31, ENSG00000248771) in publicly available datasets derived from RNA-Seq analyses performed in human colon biopsies from healthy controls and patients affected by ulcerative colitis (UC; GSE128682 datasets) was statistically analyzed using Wilcoxon test. To investigate the expression of *EPR* targets in human colon adenocarcinoma samples, we interrogated publicly available proteomic datasets from the Clinical Proteomic Tumor Analysis Consortium (CPTAC, NIH) using the interactive web resource UALCAN (25).

## RESULTS

### Conditional targeted deletion of *EPR* in mouse large intestine

*EPR* expression is prominent in the large intestine of both human and mouse (10). We analyzed the distribution of *EPR* and found that it is present in the cytoplasm, nucleoplasm and chromatin (with a prevalence in the chromatin compartment) of crypts purified from mouse large intestine (Supplementary Figure S1A).

Analysis of single cell RNA-sequencing datasets (24) revealed that *EPR* is predominantly expressed in goblet cells (GCs) while it is almost undetectable in enteroendocrine and tuft cells of the large intestine (Supplementary Figure S1B). Together with our previous observations (10), this evidence supports the notion that *EPR* is expressed in select lineages within epithelial tissues.

To abrogate *EPR* expression in colon, we first targeted the promoter and exon 1 of the gene by inserting two loxP sites at the 5′ and 3′ ends of the targeted region (Supplementary Figure S1C). Upon selection of several independent ES cell clones through Southern blot analysis (Supplementary Figure S1D), we obtained founder mice heterozygous for *EPR* gene targeting (*EPR* fl/+). Next, we utilized a well-characterized transgenic mouse line in which the CDX2 promoter drives the expression of CRE recombinase in the epithelium of the distal ileum, cecum and throughout the colon from the crypt base to the luminal surface (CDX2-CRE) (13). The choice of CDX2 regulatory regions was guided by the evidence that this gene is expressed throughout the same cell population that expresses *EPR* in the large intestine. After three rounds of crossing, we obtained a population of *EPR* fl/fl mice expressing the CRE transgene (*EPR* cKO). An example of the PCR-based genotyping strategy that we adopted is shown in Supplementary Figure S1E. As expected based on the expression of the CDX2 transgene, we obtained an almost complete ablation of *EPR* in both the distal ileum and colon of *EPR* cKO mice (Supplementary Figure S1F).

*EPR* cKO mice develop normally, are viable, fertile and are born at the expected Mendelian frequency (data not shown). Further, *EPR* cKO mice show no major morphological anomalies or functional impairment throughout adulthood, and the gross morphology of 6- to 48-week-old mice reveal no macroscopic differences between *EPR* fl/fl and *EPR* cKO mice in major organs, including lungs, heart, liver, stomach, jejunum, ileum and spleen (data not shown). However, even though knockout mice do not display overt diarrhea, we consistently noticed a reduction of formed fecal pellets in the colon of *EPR* cKO mice (Supplementary Figure S1G). In mice, the proximal colon mucus encapsulates the fecal matter, and specific alterations of the proximal colon in *EPR* cKO mice could explain this observation (see below).

**Figure 1.**
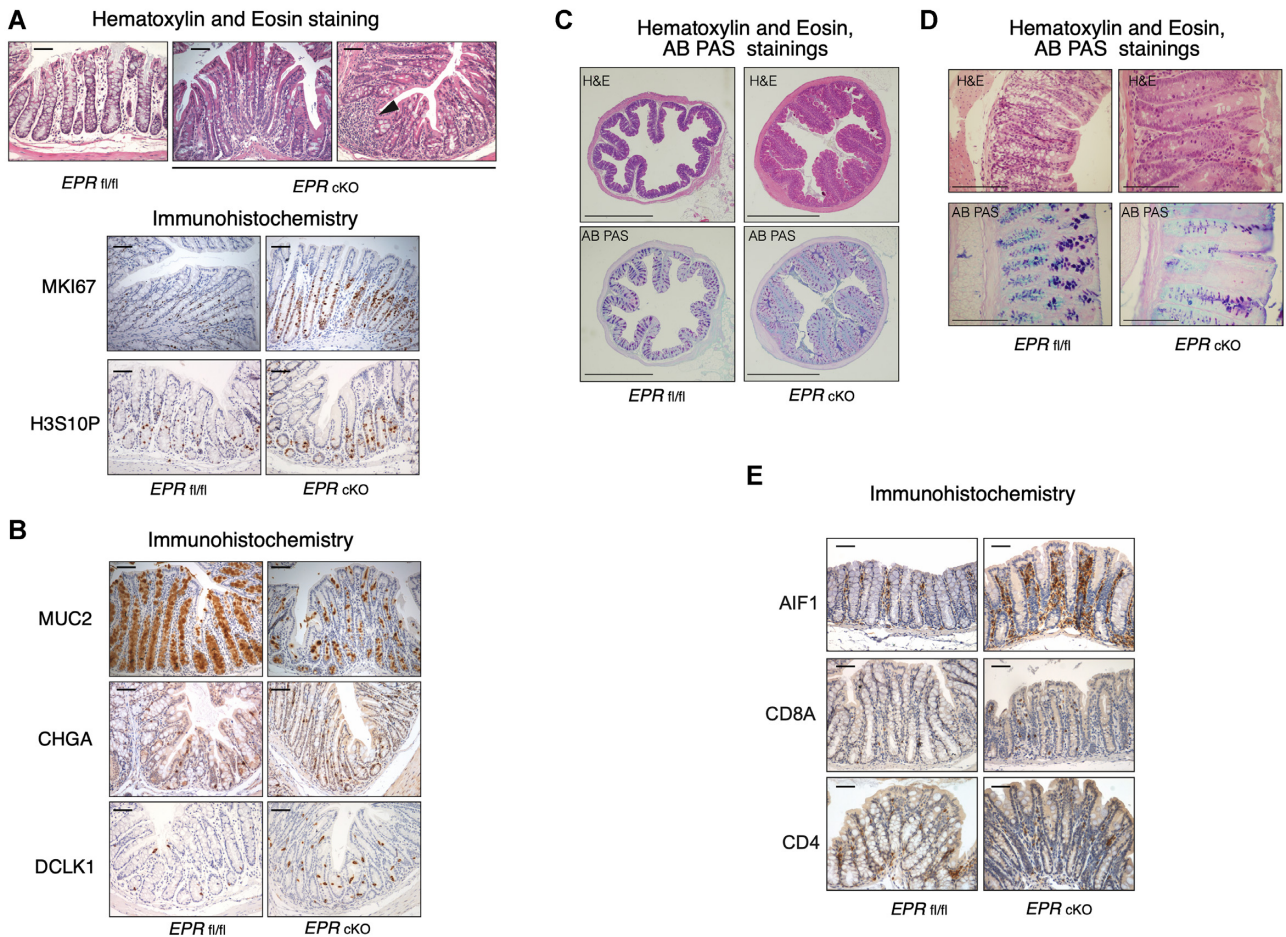
Histological and immunohistochemical analyses of colon from *EPR* cKO versus *EPR* fl/fl mice. (**A**) Upper panel, H&E staining of *EPR* fl/fl and *EPR* cKO proximal colon sections; the arrowhead indicates a lymphoid patch. Lower panel, immunohistochemical analysis of proliferation markers in *EPR* fl/fl and *EPR* cKO proximal colon sections, as indicated. Magnification ×10, scale bar 100 μm. (**B**) Immunohistochemical analysis of secretory cell markers, as indicated. Magnification ×10, scale bar 100 μm. (**C**) Upper panels, H&E staining of *EPR* fl/fl and *EPR* cKO proximal colon sections; lower panels, AB PAS staining of *EPR* fl/fl and *EPR* cKO. Magnification ×2, scale bar 1000 μm. (**D**) Upper panels, H&E staining of *EPR* fl/fl and *EPR* cKO proximal colon sections; lower panels, AB PAS staining of *EPR* fl/fl and *EPR* cKO. Magnification ×40, scale bar 200 μm. (**E**) Immunohistochemical analysis of T cells and macrophages, as indicated. Magnification ×10, scale bar 100 μm. Immunohistochemistry experiments presented in A (lower) and B were acquired, positive cells for each field were counted and the number of positive cells/crypt is presented in Supplementary Figure S2.

Histological analysis of H&E-stained sections along the proximal–distal axis of the colon revealed the presence of lymphoid patches and areas of mucosal thickening suggestive of areas of hyperproliferation (Figure 1A, upper panel). Indeed, immunohistochemical analysis revealed increased levels of two distinct proliferation markers, MKI67 and H3S10P, in the crypts of *EPR* cKO mice. (Figure 1A, lower panel; Supplementary Figure S2A). To investigate if *EPR* deletion affects the distribution of cell subpopulations in the colon epithelium, we performed further immunohistochemical analyses. As shown in Figure 1B and Supplementary Figure S2B and C, *EPR* cKO mice display a strong reduction of Mucin 2 (MUC2)-positive cells accompanied by a small but significant increment of Chromogranin A (CHGA)- and Doublecortin-like Kinase1 (DCLK1)-expressing cells. MUC2 is specifically expressed in GCs, the cell population responsible for mucus production, while CHGA and DCLK1 mark enteroendocrine and tuft cells, respectively (26). Mucins are the building blocks of the mucus layer and MUC2 is the major secreted mucin in the colon epithelium (27). AB PAS staining presented in Figure 1C and D demonstrates a strong reduction of the mucus in *EPR* cKO crypts. We questioned whether the decreased levels of MUC2-positive cells and mucus production as seen with AB PAS stain is caused by a reduced number of GCs. qRT–PCR analysis of crypts isolated from either *EPR* fl/fl or *EPR* cKO colon tissue revealed similar expression levels of *Tff3* and *C1galt1—*which are both products of fully differentiated GCs (28,29)—thus suggesting that GCs are still present in *EPR* cKO large intestine (Supplementary Figure S2D, see also below). Interestingly, we noticed that the differences in *Muc2* and mucus levels between *EPR* fl/fl and *EPR* cKO mice are more evident in the proximal third of the organ (Supplementary Figure S2E).

Alterations of intestinal mucus are known to favor inflammation (27,29) and, indeed, levels of macrophage (AIF1) and T-lymphocyte (CD8A and CD4) markers are higher in *EPR* cKO colon than in that of control mice (Figure 1E).

Altogether our data indicate that *EPR* cKO mice display hyperproliferation, impaired mucus production and inflammatory infiltration in the proximal portion of the large intestine.

### *EPR* cKO mice display strong reduction of goblet cell-specific factors including those involved in *O*-linked oligosaccharide processing

**Figure 2.**
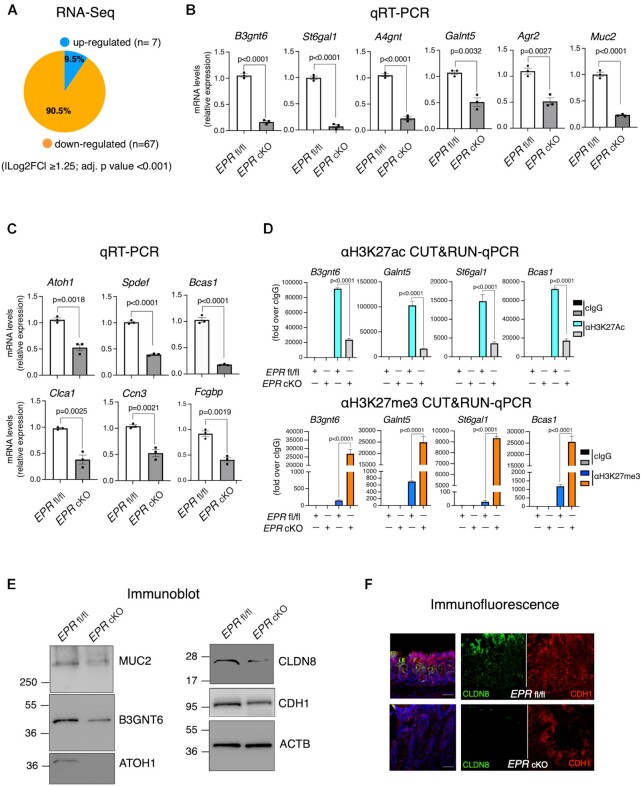
*EPR* knockout in colon affects gene expression by strongly reducing goblet cell-specific factors. (**A**) RNA-Seq analysis (sextuplicates) in the proximal colon of *EPR* cKO versus *EPR* fl/fl mice. Pie diagram shows the percentage of gene expression changes. Transcripts displaying |log2 fold expression difference| ≥1.25 (*P* <0.001, Student's *t*-test) are presented. (**B** and **C**) Total RNA was extracted from crypts purified from the proximal colon of *EPR* cKO and *EPR* fl/fl mice (as indicated). qRT–PCR analysis was performed using transcript-specific primers (listed in Supplementary Table S2). The values are averages (±SEM) of three independent experiments performed in triplicate. Statistical significance (Student's *t*-test) was calculated using GraphPad Prism 9 for macOS, and is indicated. (**D**) Crypts purified from the proximal colon of *EPR* cKO and *EPR* fl/fl mice were subjected to CUT&RUN experiments (biological duplicates) and extracted DNA was subjected to qPCR analysis using primers specific for the promoter regions of the indicated genes (see Supplementary Table S2). (**E**) Total extracts were prepared from crypts purified from the proximal colon of *EPR* cKO and *EPR* fl/fl mice and analyzed by sodium dodecylsulfate–polyacrylamide gel electrophoresis (SDS–PAGE) followed by immunoblot as indicated. (**F**) Immunofluorescence analysis of the proximal colon of *EPR* cKO and *EPR* fl/fl mice. Sections were stained with the indicated antibodies. Scale bars represent 50 μm. Data displayed are representative of at least five fields evaluated in two biological replicates.

To obtain information on *EPR* function in the large intestine epithelium, we analyzed gene expression perturbations consequent to *EPR* ablation by performing RNA-Seq in the proximal colon. As expected based on our previous observations that *EPR* can be viewed as a gene expression activator (10,11), we found that most of the differentially expressed genes are down-regulated in *EPR* cKO colon compared with controls (Figure 2A). Gene Ontology analysis of down-regulated genes showed enrichment of genes coding for factors implicated in *O*-linked oligosaccharide (*O*-glycan) processing and epithelial structure maintenance of GCs (Supplementary Figure S3A). *O*-Glycans are the major modifiers of Ser and Thr residues within mucins. Indeed, qRT–PCR analysis of RNA isolated from purified crypts of proximal large intestine showed highly significant reduction of factors involved in the metabolism of *O*-glycans besides *Muc2* itself in *EPR* cKO mice (Figure 2B). Further, *EPR* cKO mice display reduced expression of several additional factors that are either GC specific or enriched in this cell population (Figure 2C; Supplementary Figure S3B).

We have previously reported that *EPR* overexpression in a mammary gland cell line results in accumulation of histone marks of gene transcriptional activation at the promoter region of *EPR* targets (11). We showed by CUT&RUN-qPCR analyses that the accumulation of the gene transcription activation mark histone H3 acetylated lysine 27 (H3K27ac) is significantly reduced at the promoter of several *EPR*-regulated promoters while the amount of the histone H3 trimethylated lysine 27 (H3K27me3) repression mark is strongly enhanced at the same promoters in crypts from *EPR* cKO mice (Figure 2D).

Importantly, immunoblot and immunofluorescence analyses presented in Figure 2E and F, respectively, show the reduction of GC-specific factors—as well as of factors involved in cell adhesion—in *EPR* cKO colon crypts in comparison with controls. As expected based on CDX2-CRE transgene expression (13), *EPR* is also abrogated in the terminal ileum and, consequently, the expression of some GC-enriched transcripts is down-regulated in the ileum of *EPR* cKO mice (Supplementary Figures S1F and S3C). Considering that some lncRNAs can affect the expression of neighboring genes *in cis* (2,3), we investigated this possibility by RNA-Seq and qRT–PCR. As shown in Supplementary Figure S4, the expression of genes at both the 5′ and 3′ end of *EPR* is not affected by its conditional deletion.

Altogether, our data indicate that *EPR* cKO causes a rearrangement of the colon crypt transcriptome with a prominent down-regulation of genes coding for factors expressed in GCs and involved in the metabolism of *O*-glycans.

### Ultrastructural features and permeability alterations in the intestinal mucosa of *EPR* cKO mice

**Figure 3.**
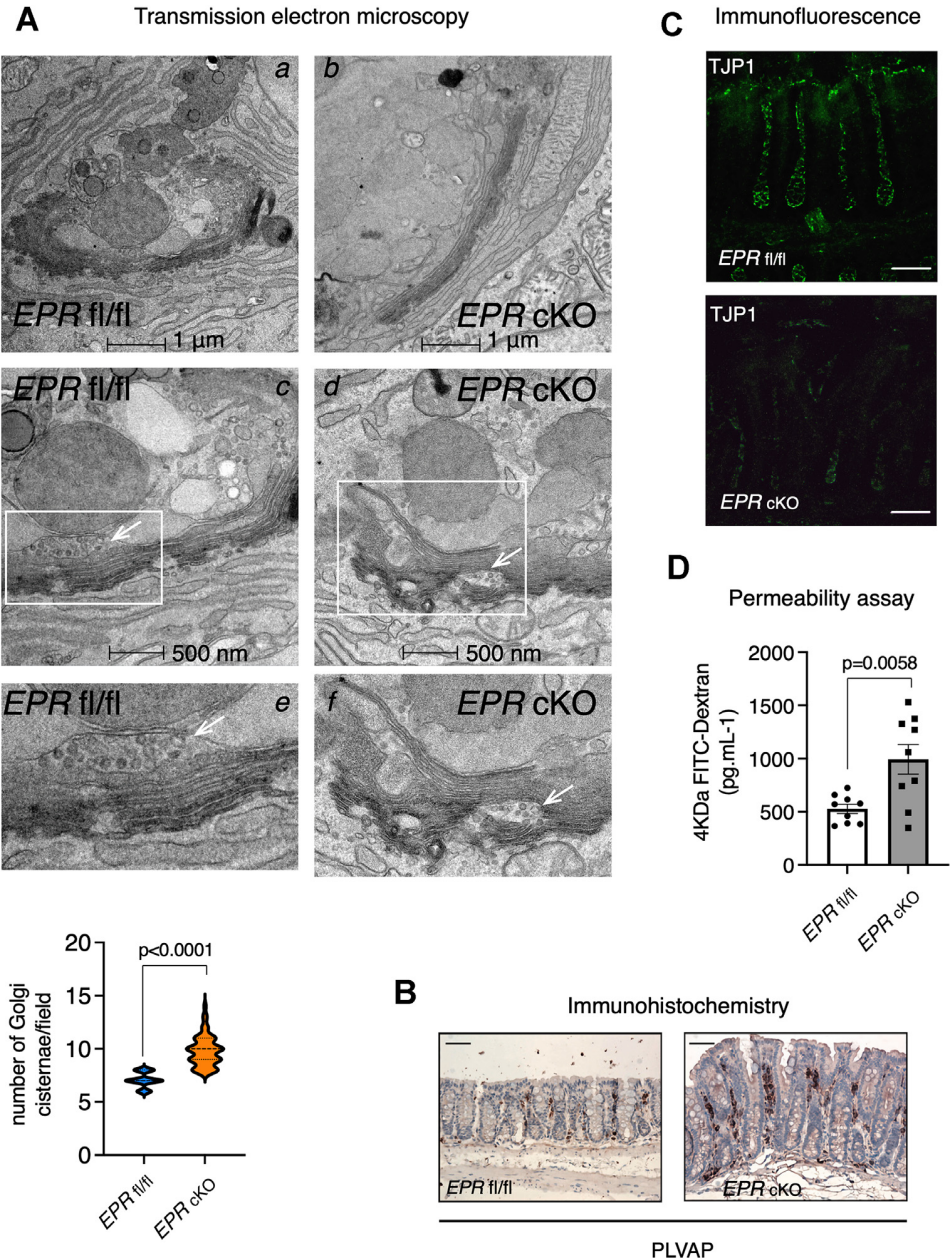
Ultrastructural features and permeability alterations in the intestinal mucosa of *EPR* cKO mice. (**A**) TEM analysis of GCs in the proximal colon of *EPR* cKO and *EPR* fl/fl mice (as indicated, three biological replicates per condition). Panels e and f represent enlargement of the areas delimitated by white boxes in panels c and d, respectively. Arrows in panels c, d, e and f point to post-Golgi vesicles whose number is reduced in *EPR* cKO mice. Scale bars are indicated below images. Quantification in graph panel. The number of Golgi cisternae was counted in 20 distinct sections per each biological replicate, and the statistical significance of differences among *EPR* fl/fl and *EPR* cKO mice was analyzed using Student's *t*-test. (**B**) Immunohistochemical analysis of plasmalemma vesicle-associated protein (PLVAP) distribution in *EPR* fl/fl and *EPR* cKO colon. Magnification ×10, scale bar 100 μm. (**C**) Immunofluorescence analysis (representative of at least five fields evaluated in two biological replicates) of the proximal colon of *EPR* fl/fl and *EPR* cKO mice. Sections were stained with anti-TJP1 antibody. Scale bars represent 50 μm. (**D**) Serum concentrations of FITC–dextran in *EPR* cKO and *EPR* fl/fl mice (as indicated, nine biological replicates) were measured 4 h after oral administration of FITC–dextran. Statistical significance (Student's *t*-test) has been calculated using GraphPad Prism 9 for macOS, and is indicated.

To obtain more insights into the morphological features of *EPR* cKO intestinal mucosa, we performed TEM. At the ultrastructural level, GCs display a high heterogeneity, with the presence of cells with normal features intermingled with cells displaying abnormalities. As presented in Figure 3A, abnormal GCs show a higher number of Golgi cisternae (two additional cisternae, on average, see quantification in the graph panel) that are also less isolated. Further, mucus is homogeneously less osmiophilic in *EPR* cKO GCs, and secretory granules fuse with each other via membrane breaks more often in GCs from *EPR* cKO than in GCs from *EPR* fl/fl control mice. Finally, the number of clathrin-dependent vesicles in the area of secretory granules is lower in *EPR* cKO GCs than in controls cells.

These data suggest that the Golgi complex in *EPR* cKO GC is impaired, and this is associated with reduced mucus production.

Considering the histochemical and ultrastructural features of colon mucosa in *EPR* cKO mice, we set out to investigate if *EPR* ablation affects mucosa integrity and intestinal permeability. First, we analyzed the expression of plasmalemma vesicle-associated protein (PLVAP), an endothelial cell-specific factor which is considered a marker of endothelial barrier permeability (30). Immunohistochemical analysis in Figure 3B shows enhanced levels of PLVAP in the intestinal mucosa of *EPR* cKO mice. Further, the expression of TJP1—a tight junction scaffold protein reduced in inflammatory bowel disease (IBD) (31)—is down-regulated in *EPR* cKO mice (Figure 3C). To evaluate and quantitate intestinal permeability, we fed both control (*EPR* fl/fl) and *EPR* cKO mice with FITC–dextran by gavage and measured FITC–dextran serum levels after 4 h. Figure 3D shows that FITC–dextran serum levels are significantly higher in *EPR* cKO mice than in *EPR* fl/fl mice.

Collectively, these data indicate that colon mucosa integrity and permeability are impaired in *EPR* cKO mice.

### *EPR* cKO mice are highly susceptible to DSS-induced colitis and tumor formation

**Figure 4.**
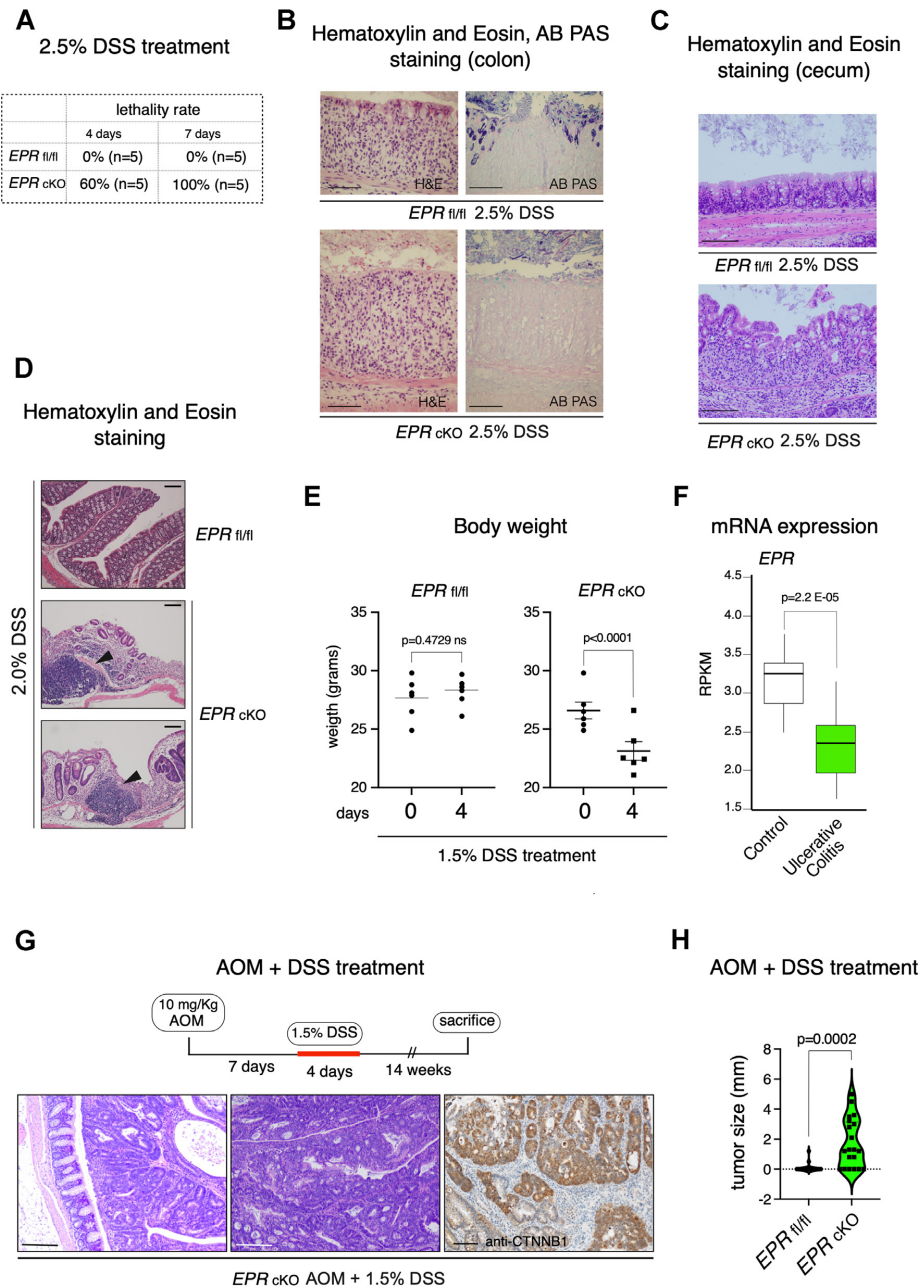
*EPR* cKO mice are highly susceptible to DSS-induced colitis and tumor formation. (**A**) Lethality rate of a cohort of five age-matched *EPR* cKO and *EPR* fl/fl mice after 4 and 7 days of treatment with 2.5% DSS dissolved in drinking water. (**B**) AB PAS staining of *EPR* fl/fl and *EPR* cKO proximal colon sections after a 7 day treatment with 2.5% DSS dissolved in drinking water, magnification ×20, scale bars 100 μm. (**C**) AB PAS staining of *EPR* fl/fl and *EPR* cKO proximal colon sections after a 7 day treatment with 2.5% DSS dissolved in drinking water, magnification ×20, scale bars 200 μm. (**D**) H&E staining of colon sections from *EPR* fl/fl and *EPR* cKO mice treated for 5 days with 2% DSS; arrowheads indicate immune infiltrate. Magnification ×10, scale bar 100 μm. (**E**) Weight loss in a cohort of six *EPR* cKO mice compared with six age-matched *EPR* fl/fl mice after a 4 day treatment with 1.5% DSS dissolved in drinking water. Statistical significance (Student's *t*-test) was calculated using GraphPad Prism 9 for macOS, and is indicated. (**F**) Expression analysis of *EPR* in UC samples and in normal controls. The GSE128682 datasets were analyzed and reads per kilobase million (RPKM) corresponding to human *EPR* were statistically analyzed using Wilcoxon test. (**G**) Upper panel, schematic of the sequential treatment with AOM and DSS. Lower left and middle panels, H&E staining of representative tumors developed in *EPR* cKO mice (magnification ×10, scale bars 200 μm); right panel, immunohistochemical analysis of β-catenin (CTNNB1) in a tumor sample; magnification ×10, scale bars 100 μm. (**H**) Size of tumors detected in the large intestine of a cohort of mice subjected to the treatment described above. Statistical significance (Student's *t*-test) was calculated using GraphPad Prism 9 for macOS, and is indicated.

DSS is a heparin-like polysaccharide that, dissolved in drinking water, induces colon epithelium damage and colitis in mice, mimicking some features of IBD (14). We used this model to assess the response of *EPR* cKO mice to an acute pro-inflammatory challenge. Pilot experiments aimed at evaluating the response of mice to DSS revealed high susceptibility of *EPR* cKO to 2.5% DSS, with diarrhea, weight loss and fecal bleeding more evident than in control mice (data not shown). Importantly, we observed 60% and 100% lethality in *EPR* cKO mice after 4 and 7 days of treatment, respectively (compared with no lethality in control *EPR* fl/fl mice) (Figure 4A). Histological analysis of the colon and cecum revealed more severe crypt disruption, massive inflammatory cell infiltration in the tissue and reduction of AB PAS-positive cells in *EPR* cKO when compared with *EPR* fl/fl (Figure 4B, C). As shown in Figure 4D, *EPR* cKO mice treated with 2% DSS also display areas of severe inflammatory infiltration when compared with *EPR* fl/fl control mice. Given the high lethality induced by DSS in *EPR* cKO mice, we designed a new treatment protocol by reducing the DSS concentration to 1.5% and the treatment duration to 4 days. Even under these conditions, a significant reduction of body weight was evident in *EPR* cKO mice while no changes in body weight were detectable in *EPR* fl/fl mice (Figure 4E).

Based on the above observations, we set out to analyze the expression of human *EPR* (LINC01207 aka SMIM31, ENSG00000248771) in publicly available datasets derived from RNA-Seq analyses performed in human colon biopsies from healthy controls and patients affected by UC. Figure 4F shows that UC-affected patients display a significantly reduced expression of human *EPR* in their colon.

It is known that patients with UC exhibit an increased risk of colorectal cancer (32), and we explored the possibility that *EPR* cKO mice are more susceptible to colorectal tumorigenesis. *EPR* cKO and *EPR* fl/fl mice were intraperitoneally injected with AOM and, after 1 week, treated with 1.5% DSS in drinking water for 4 days. After 14 weeks, mice were analyzed (schematic displayed in the top panel of Figure 4G). Only *EPR* cKO mice developed macroscopically evident tumors (40%, data not shown). The histological analysis revealed that 56% of *EPR* cKO mice developed multiple adenomas, with dysplasia ranging from low to high grade (examples provided in Figure 4G, left panel) compared with 29% of *EPR* fl/fl mice. Figure 4H shows the size distribution of colon adenomas in *EPR* cKO and control mice.

Taken together, our data indicate that *EPR* cKO mice are more prone to severe inflammation and tumor formation than control mice.

### *EPR* is down-regulated in human cancer cell lines and in human cancers

**Figure 5.**
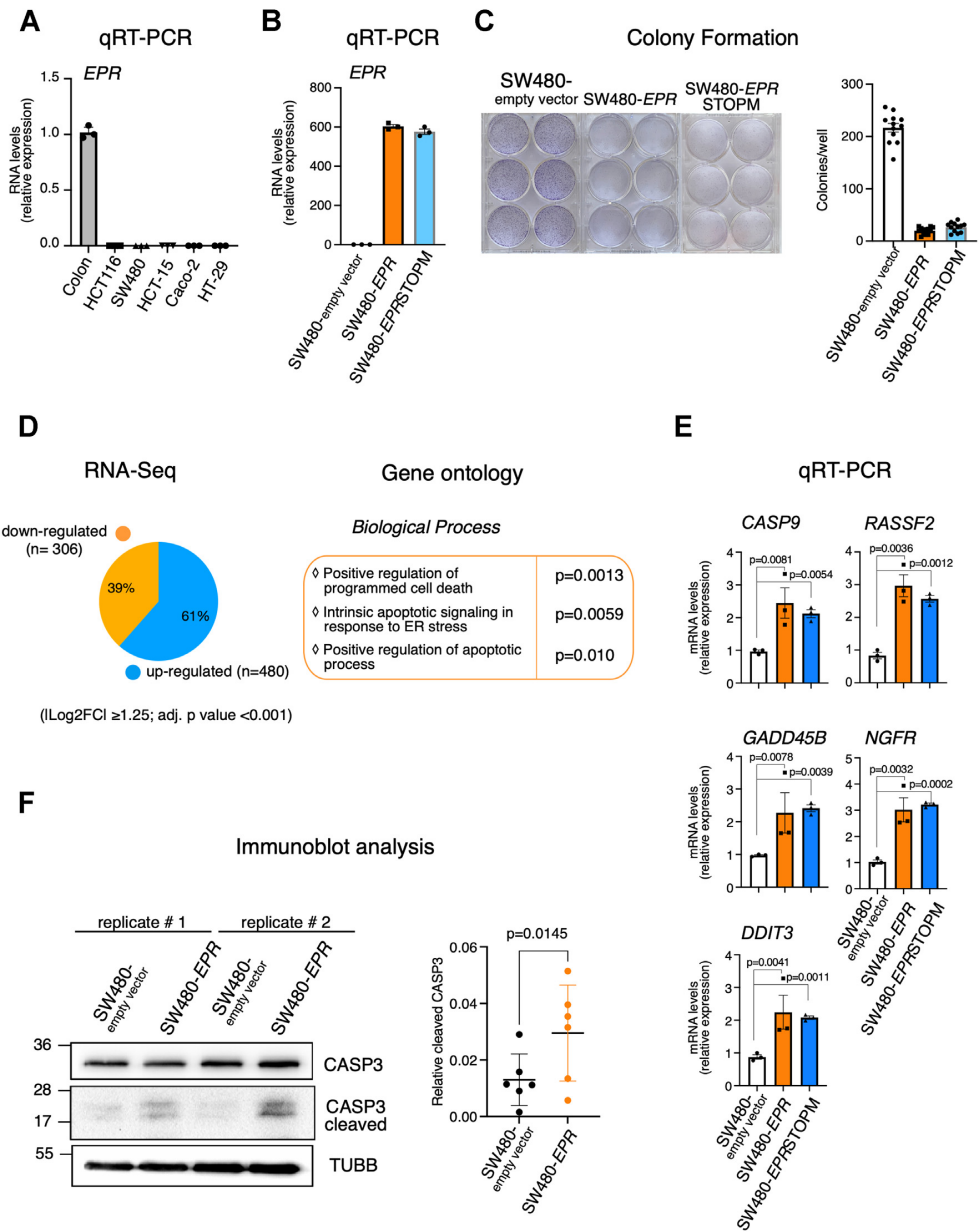
*EPR* is down-regulated in cancer cell lines and its expression induces pro-apoptotic genes. (**A**) The expression of *EPR* was quantified by qRT–PCR analysis in the indicated cell types. The values of qRT–PCR are averages (±SEM) of three independent experiments performed in triplicate. (**B**) Total RNA was extracted from SW480-empty vector, SW480-*EPR* and SW480-*EPR*STOPM cells, and EPR expression was analyzed by qRT–PCR. Results are averages (±SEM) of three independent experiments performed in triplicate. (**C**) SW480-empty vector, SW480-*EPR* or SW480-*EPR*STOPM cells were plated at low density in sextuplicate. After 6–10 days, colonies were stained and counted using the imaging analysis software package ImageJ 1.53a (http://imagej.nih.gov/ij, NIH). On the left is shown a representative image of stained colonies. The values of three independent experiments performed in sextuplicate (±SEM) are plotted on the right. (**D**) Left panel: RNA-Seq analysis (performed in triplicate) in SW480-*EPR* versus SW480-empty vector cells. Pie diagram showing the percentage of gene expression changes. Transcripts displaying |log2 fold expression difference| ≥1.25 (*P* <0.001, Student's *t*-test) are presented. Right panel: Gene Ontology biological process analysis (using the online EnrichR tool) of transcripts whose levels are affected by *EPR* expression in SW480 cells. (**E**) qRT–PCR analysis of the indicated transcripts in SW480-empty vector, SW480-*EPR* or SW480-*EPR*STOPM cells. The values are averages (±SEM) of three independent experiments performed in triplicate. Statistical significance (Student's *t*-test) was calculated using GraphPad Prism 9 for macOS, and is indicated. (**F**) Left panel: total extracts from SW480-empty vector and SW480-*EPR* cells were analyzed by SDS–PAGE followed by immunoblot as indicated. The immunoblots displayed are two replicates and are representative of three independent experiments performed in duplicate that yielded similar results. Right panel: quantification of the relative cleaved caspase 3 expression levels. Statistical significance (Student's *t*-test) was calculated using GraphPad Prism 9 for macOS, and is indicated.

We investigated the expression of human *EPR* in several human cancer cell lines and found that the lncRNA is barely detectable when compared with normal human colon (Figure 5A). Based on this observation, we decided to ectopically express *EPR* in the colorectal adenocarcinoma cell line SW480 (SW480-*EPR*, Figure 5B). We previously reported that *EPR* contains a short ORF able to encode a small peptide that has very limited effect on the gene expression changes induced by *EPR* overexpression in mammary gland cells (10). Thus, we wanted to verify the impact of the peptide in colon cancer cells by also transfecting SW480 with an *EPR* mutant in which the start codon of the peptide has been mutagenized to a STOP codon (SW480-*EPR*STOPM, Figure 5B). Preliminarily, we fractionated SW480-*EPR* and SW-480-*EPR*STOPM, and analyzed the subcellular distribution of transfected *EPR*. Data presented in Supplementary Figure S5A show that wild-type and mutant transfected *EPR* share a similar subcellular localization with endogenous *EPR* in colon crypts. Next, we observed that SW480-*EPR* cells show a significantly lower ability to form colonies in comparison with empty vector-transfected SW480 cells (SW480-empty vector, Figure 5C). Importantly, *EPR*STOPM induces a comparable reduction of the colony formation ability (Figure 5C). As expectable based on our previous observations (10 and this report), *EPR* expression in SW480 induces a vast rearrangement of the transcriptome as revealed by RNA-Seq analysis, with two-thirds of the genes being up-regulated by the lncRNA (Figure 5D, left panel). Gene Ontology analyses of up-regulated genes revealed the enrichment of terms related to the apoptotic process (Figure 5D, right panel). qRT–PCR analysis presented in Figure 5E validated the significant induction of pro-apoptotic transcripts in SW480-*EPR-* and in *EPR*STOPM-expressing SW480 cells. Finally, Figure 5F shows that the percentage of cleaved caspase 3 is enhanced in SW480-*EPR*- compared with empty vector-transfected cells. Altogether these data indicate that *EPR* expression in colon adenocarcinoma cell lines induces genes involved in the apoptotic process.

**Figure 6.**
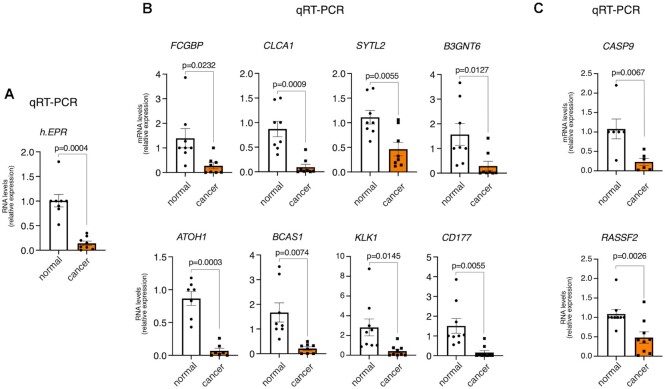
*EPR* and select target genes are down-regulated in human cancers. The expression of *EPR* (**A**) and of a selection of its target genes in mice (**B**) or in SW480 cells (**C**) was analyzed by qRT–PCR in colon adenocarcinomas and in the adjacent normal tissue. Statistical significance (Student's *t*-test for paired data) was calculated using GraphPad Prism 9 for macOS, and is presented.

Next, we investigated the expression of *EPR* in a group of human colon adenocarcinoma tissues and in the corresponding normal tissue. qRT–PCR analysis showed that *EPR* is significantly down-regulated in cancers (Figure 6A). Since conflicting results on human *EPR* expression in colorectal cancers exist in the literature (33,34), we confirmed *EPR* down-regulation by using four sets of primers spanning distinct regions of the lncRNA (Supplementary Figure S5A). Interestingly, we observed that the levels of proteins encoded by genes down-regulated in the large intestine of the *EPR* cKO mice are reduced in a cohort of primary colon adenocarcinomas as revealed by the analysis of datasets from the Clinical Proteomic Tumor Analysis Consortium (CPTAC; Supplementary Figure S5B). Most importantly, we found that some genes down-regulated in colon crypts of *EPR* cKO mice (Figure 6B) and some transcripts up-regulated in SW480-*EPR* (Figure 6C) are severely down-regulated in cancers when compared with adjacent normal tissue.

Altogether, these data suggest a potential role for *EPR* in human colorectal carcinogenesis.

### EPR directly interacts with select target genes and its knockout affects chromatin long-range promoter interactions

**Figure 7.**
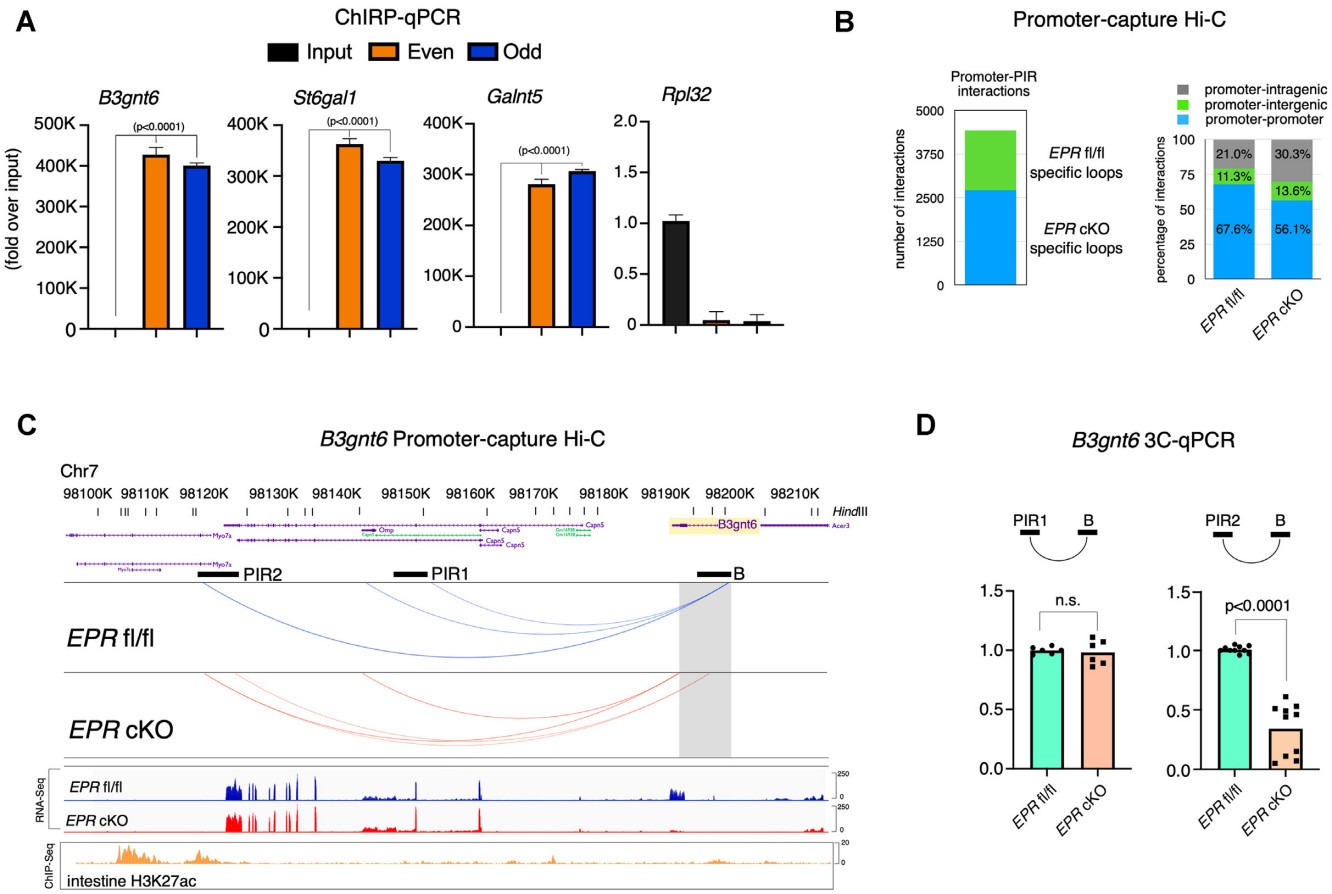
EPR–chromatin interaction and remodeling of 3D chromatin circuitry in colon crypts upon *EPR* knockout. (**A**) qPCR analysis of *EPR* genomic targets. Both input DNA and DNA purified using either ODD (red bars) or EVEN (blue bars) tiling oligonucleotides were analyzed by qPCR to amplify a promoter region in the indicated target genes or in the *Rpl32* gene (negative control). Values, represented as fold increase over input, are averages (±SEM) of three independent experiments performed in triplicate. (**B**) Bar graph displaying the number of specific promoter–PIR interactions in colon crypts from *EPR* fl/fl and *EPR* cKO mice as indicated (left panel), and the proportion of interactions of promoters with PIRs as indicated (right panel). (**C**) WashU EpiGenome Browser snapshot showing the interactions between the *B3gnt6* promoter region and two distinct PIRs. The shaded area corresponds to the promoter region present in the Arima promoter panel. Two interacting genomic regions have been designed PIR1 and PIR2, respectively, and analyzed in 3C experiments (see D). The bait region is indicated as B. Snapshots of RNA-Seq analysis are provided below (as indicated) as well as a snapshot of H3K27ac ChIP-seq in intestine (GSM2192070 ENCODE). (**D**) Schematic of promoter–PIR interactions analyzed (top panel), 3C analysis performed on either *EPR* fl/fl or *EPR* cKO colon crypts (bottom panel). DNA fragments obtained upon HindIII digestion and ligation were analyzed by qPCR. The values of qRT–PCR experiments shown are averages (±SEM) of at least three independent experiments performed in triplicate. Statistical significance (Student's *t*-test) was calculated using GraphPad Prism 9 for macOS, and is indicated.

We have previously shown that EPR directly interacts with chromatin at select loci and that its binding to the *Mettl7a1* promoter favors the long-range interaction of this region with a distal enhancer element in mammary gland cells (10–12). Based on these observations, we first wanted to clarify if *EPR* can also directly interact with specific target genes in mouse colon crypts. With this aim, we performed ChIRP followed by qPCR analysis. Data presented in Figure 7A indicate that *EPR* interacts with the promoter region of transcriptionally regulated targets (*B3gnt6*, *St6gal1* and *Galnt5*) while it does not show any specific binding to a control gene. Next, we decided to comprehensively map long-range interactions between promoters and distal regulatory elements that might be affected by *EPR* knockout in the large intestine. For this purpose, we performed promoter capture Hi-C (PC-Hi-C) experiments. We enriched Hi-C libraries prepared from crypts purified from the proximal colon of *EPR* fl/fl and *EPR* cKO mice for promoter interactions through hybridization with biotinylated RNA probes from the Arima mouse promoter panel (designed to hybridize promoters of 25 752 mouse genes; see the Materials and Methods). We used the Arima pipeline—that includes pre-processing of data using HiCUP (21) and loop calling using CHiCAGO (22)—to assign confidence scores to the interactions between the captured promoter fragments and the promoter-interacting regions (PIRs). We identified 40 911 promoter interactions in *EPR* fl/fl and 58 494 in *EPR* cKO. A large proportion of interactions (∼90%) are shared between the two conditions while *EPR* knockout specifically affects 4420 interactions [false discovery rate (FDR) of the looping interactions <1.0E-5, logFC ≥6.0, Figure 7B, left panel]. The total number of contacts of each promoter with PIRs is not affected by *EPR* knockout (mean 1.28 in *EPR* fl/fl and 1.34 in *EPR* cKO). Approximately 68% of the *EPR* fl/fl-specific interactions and 56% of the *EPR* cKO-specific interactions are between two promoter regions, while the remaining interactions are between promoters and intergenic or intragenic regions (Figure 7B, right panel). Non-homologous interchromosomal contacts have been shown to contribute to transcriptional regulation (35), but analysis of specific contacts in *EPR* fl/fl and *EPR* cKO indicates that all the observed interactions occur within the same chromosome (data not shown). The highest number of interactions (normalized per Mb of chromosome length) are in chromosomes 11 and 7 (Supplementary Figure S6A), a figure that is independent of gene density (https://genome.ucsc.edu/cgi-bin/hgTracks?db=mm10&chromInfoPage =), and the normalized number of loops per chromosome is similar between *EPR* fl/fl and *EPR* cKO (Supplementary Figure S6A). The median distance between promoters and PIRs is ∼100 kb in both *EPR* fl/fl and *EPR* cKO (Supplementary Figure S6B). Interestingly, the distance distribution between promoters and PIRs significantly differs between *EPR* fl/fl and *EPR* cKO crypts (*P*-value = 2.198e-14, Student's *t*-test) with shorter interactions (≤100 kbp) being favored in *EPR* cKO and longer interactions (≥300 kbp) in *EPR* fl/fl crypts (Supplementary Figure S6B). Next, we conducted motif analysis on promoters that interact with PIRs and on PIRs themselves in either *EPR* fl/fl or *EPR* cKO using the HOMER tool (http://homer.ucsd.edu/homer/motif/). We observed that distinct motifs are over-represented in *EPR* fl/fl and *EPR* cKO, respectively, and this finding allows us to hypothesize that *EPR* selectively controls the 3D genomic organization cooperating with specific transcription factors (Supplementary Figure S6C, D).

Next, we investigated whether the 3D chromatin remodeling observed in colon crypts in *EPR* cKO is correlated with the gene expression changes that we described in this study (see Figure 2 and Supplementary Figure S3). We focused our analysis on a set of genes that encode factors involved in the regulation of mucus production and whose expression is strongly reduced in *EPR*-deleted colon crypts. As shown in Figure 7C, the *B3gnt6* promoter interacts with two distinct distal elements (PIR1 and PIR2 in Figure 7C) in *EPR* fl/fl. *EPR* knockout abrogates these interactions while it favors the interaction of a distinct region of the *B3gnt6* gene (first intron 3′ to a short untranslated exon) with two distinct PIRs that are adjacent to those contacting the *B3gnt6* promoter in *EPR* fl/fl (Figure 7C). As a validation of PC-Hi-C experiments, 3C-qPCR analysis showed that the interaction of the bait region of *B3gnt6* with PIR2 is severely affected by *EPR* knockout (Figure 7D). Further, the results displayed in Supplementary Figure S7 show that different patterns of promoter–PIR(s) interaction occurs in *Galnt7*, *Fut8*, *Clca1* and *Ccn3* genes because of *EPR* knockout.

Altogether, our data point to a reorganization of long-range promoter interactions in colon crypts upon *EPR* conditional ablation.

## DISCUSSION

Here, we report an investigation on the physiological role of *EPR* through the conditional generation and characterization of an *EPR-*deficient mouse line in the colon.

*EPR* cKO mice display altered mucus structure and function, impaired colon mucosa integrity and increased intestinal permeability, as well as high susceptibility to chemical-induced colitis and colorectal tumorigenesis. Mechanistically, *EPR* interacts with chromatin at select genes that are transcriptionally down-regulated upon its knockout. Further, EPR deletion is accompanied by rearrangements of the long-range interactions between promoters and putative regulatory elements in colon crypts.

We decided to use a conditional approach because *EPR* is expressed in most epithelial tissues (10) and its complete deletion could have affected mice development and/or viability. Our past and present results indicate that the expression of *EPR* is restricted to specific highly differentiated cells within epithelial tissues. In human mammary gland, we have shown that *EPR* is expressed in differentiated luminal cells (10) and now we show that, in the intestine, mainly GCs are affected by its deletion. According to published scRNA-Seq data (24), *EPR* is also present, though at a lower level, in enterocytes. However, specific enterocyte markers [e.g. *Rbp2*, *Anpep* and *Fabp2* (36)] are unaffected by *EPR* deletion (our RNA-Seq results). Future scRNA-Seq analyses in *EPR* cKO mice compared with control mice will define more precisely the impact of EPR deletion on other intestinal cell lineages besides GCs.

GCs, that are central players in the regulation of essential functions during health and disease, are well known for their role in the maintenance of the colonic protective mucus barrier as well as in regulating gut immune responses (37,38). *EPR* deletion affects GC morphology and functionality by strongly reducing the expression of several genes encoding differentiated GC factors. The evidence that some specific GC markers (*Tff3* and *C1galt1*, see Supplementary Figure S2D) are still expressed in the colon of *EPR* cKO mice, together with our TEM data, suggest that GCs are present even though the expression profile of many GC-specific factors is altered, and mucus is not properly produced. Interestingly, scRNA-Seq analyses have recently unveiled notable heterogeneity within intestinal GCs which points to a markedly nuanced orchestration of the protective mucus system in the gut (38,39). Several distinct GC subpopulations endowed with specialized functions have been identified so far, and it will be interesting to investigate if all the subpopulations are similarly affected by *EPR* cKO.

Our RNA-Seq analysis demonstrates that *EPR* deletion causes a very strong reduction of several core components of the intestinal mucus (e.g. MUC2, FCGBP and CLCA1), of factors assisting synthesis, assembly, transport and control of mucus proteins (e.g. AGR2, ERN2 and SYTL2), as well as of many of the enzymatic machineries that modify the protein backbone of mucins (e.g. B3GNT6, STGAL1, GALNT7 and FUT8). *O*-Glycan-dependent modifications are the major post-translational changes of MUC2 which, in turn, is the major component of the mucus layer (38,39). Previous studies have demonstrated that two major types of *O*-glycans, named core 1 and core 3 *O*-glycans, collectively contribute to mucus layer integrity, thus preventing the unrestricted access of bacteria to the mucosa that would lead to spontaneous chronic inflammation (16,40,29). C1GALT1, expressed throughout the colon, controls the synthesis of core 1 *O*-glycans and its knockout in mice causes spontaneous colitis in the distal regions of the colon (29). Conversely, B3GNT6 is predominantly expressed in the proximal colon, regulates core 3 *O*-glycan formation and its knockout causes increased susceptibility to colitis and colorectal cancer (16). Loss of both intestinal core 1- and 3-derived *O*-glycans causes colitis in the proximal and distal regions of the colon characterized by earlier onset and more severe inflammation. *EPR* cKO mice, whose phenotype is more evident in the proximal colon where B3GNT6 is predominantly expressed, display normal levels of C1GALT1 and, as in B3GNT6 knockout, colitis is only apparent upon DSS challenge.

Interestingly, TEM analysis showed a statistically significant increased number of Golgi cisternae in GCs of *EPR* cKO mice. The number of cisternae in the Golgi apparatus is considered a stable parameter within a given cell type (41) and we propose that their increased number in *EPR* cKO mice might be the consequence of the defective maturation and secretion of mucus in the medial and in the *trans*-most Golgi cisternae (42).

The gut–vascular barrier (GVB) is the gatekeeper that controls the access of microorganisms and molecules in the systemic blood circulation (43). Our evidence of increased levels of PV1—a blood endothelial-specific factor associated with the diaphragms of the fenestrated endothelium (43)—together with enhanced total intestinal permeability, support the idea that the GVB is impaired in *EPR* cKO mice.

IBD is an umbrella term which includes chronic inflammatory conditions of the gastrointestinal tract such as UC and Crohn's disease (CD) (37). Both UC and CD are accompanied by dysregulation of mucin synthesis and altered post-translational modifications leading to barrier dysfunction. Van der Post *et al.* showed that alterations of the colon mucus barrier composition are an early event in UC pathogenesis and that structural mucus weakening occurs independent of local inflammation (44). Interestingly, most of the protein identified in the same study as mucus structural components reduced in UC are also reduced in *EPR* cKO mice (44). Interestingly, our metadata analysis indicating that human *EPR* is reduced in patients affected by UC suggests the implication of human *EPR* in this disease.

Carcinogenesis that occurs upon chronic colon inflammation has been extensively investigated and depends on different causes (45,46). The phenotype displayed by mice lacking *EPR* in the large intestine further substantiates the links between inflammation and tumorigenesis, and supports our previous hypothesis that *EPR* acts as a tumor suppressor (10). Indeed, *EPR* is significantly reduced in primary colon adenocarcinomas and, importantly, some genes down-regulated in colon crypts derived from *EPR* cKO mice are also down-regulated in human colon adenocarcinomas.

The 3D organization of the genome is linked to its function, and assigning distal regulatory regions to their target genes is crucial to understanding gene expression control (47,48). Recent advances in chromosome conformation capture technologies such as Hi-C have increased the potential to understand long-range gene control. PC-Hi-C, that includes sequence capture to pull-down fragments containing nearly all annotated promoters for a specific genome from Hi-C libraries, allows strong enrichment for promoter interactions (47,48). By applying this powerful technique to our experimental model, we found that *EPR* conditional ablation affects several interactions between promoter regions and potential distal regulatory elements. By focusing our analyses on genes whose expression is down-regulated in *EPR* cKO and exert relevant functions in mucus synthesis and modifications, we observed qualitative changes in promoter–PIR contact propensity between *EPR* fl/fl and *EPR* cKO mice. This is an interesting mechanism by which a chromatin-interacting lncRNA can affect gene expression and might lead to several future developments.

Our results represent the foundation to build up studies on specific 3D chromatin rearrangements that mediate *the EPR* protective function in colon inflammation and tumorigenesis.

## DATA AVAILABILITY

RNA deep-sequencing analyses performed in *EPR* fl/fl and *EPR* cKO colon have been published on the GEO archive under the accession GSE218994; RNA deep-sequencing analyses performed in SW480-empty vector and SW480-EPR have been published on the GEO archive under the accession GSE219181; promoter-capture Hi-C data in *EPR* fl/fl and *EPR* cKO colon crypts have been published on the GEO archive under the accessions GSE220309 and GSE220310.

## Supplementary Material

gkad257_Supplemental_File
