# Supplementary material for: LncRNA EPR regulates intestinal mucus production and protects against inflammation and tumorigenesis
Source: Nucleic Acids Res. 2023 Apr 18;51(10):5193–209. doi: 10.1093/nar/gkad257 (PMC10250242; doi:10.1093/nar/gkad257)
Supplement: gkad257_Supplemental_File [file gkad257_supplemental_file.pdf]

SUPPLEMENTARY DATA

LncRNA EPR regulates intestinal mucus production and protects against inflammation and tumorigenesis

Paola Briata, Luca Mastracci, Ettore Zapparoli, Luca Caputo, Elisa Ferracci, Alessandra Silvestri, Anna Garuti, Meriem Hadjer Hamadou, Alberto Inga, Elisa Marcaccini, Federica Grillo, Gabriele Bucci, Pier Lorenzo Puri, Galina Beznoussenko, Alexander Mironov, Fulvio Chiacchiera, Roberto Gherzi

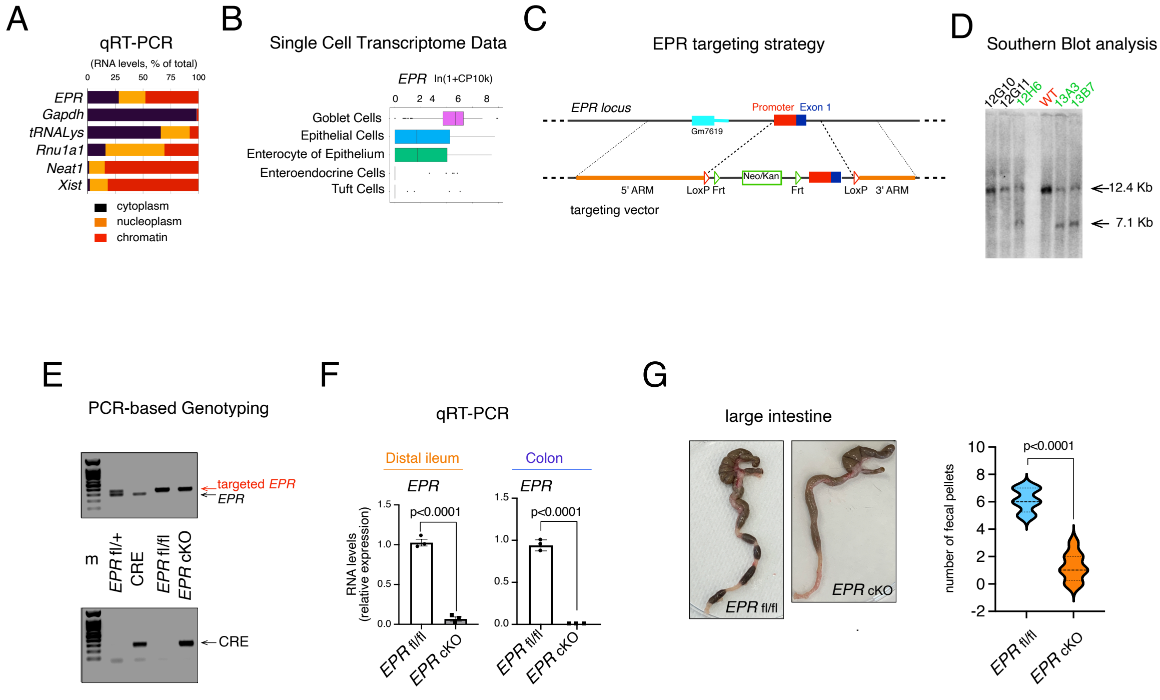

<u>Supplementary Figure S1</u>

A. Crypts from proximal colon of *EPR* fl/fl mice were fractionated and RNA was prepared from cytoplasm, nucleoplasm, and chromatin and analyzed by qRT-PCR to quantify the indicated RNAs. *Rnu1a1* is also known as U1 small nuclear RNA, *tRNALys* is the tRNA molecule that binds L-lysine. B. *EPR* expression in distinct cell types from mouse large intestine as revealed by single cell RNA-Seq according to data accessible online from The *Tabula Muris* Consortium (24). C. Schematic of the *EPR* locus targeting strategy. FRT are Flippase recombinase target sequences, LoxP are sites recognized by Cre recombinase. D. Southern blot analysis. DNA from 6 representative ES cell clones was digested using the restriction enzyme *Bam*HI. A 5' external probe was used for detection of the *EPR* alleles. DNA from C57BL/6 mice served as wild type control (WT). The wild type signal has a size of 12.4 kb while a signal of 7.1 kb represents the correctly targeted allele (as indicated by arrows). E. Representative PCR analyses of DNA extracted from ear punches of mice belonging to different colonies as indicated. F. Total RNA was prepared from distal ileum or colon (total tissue, as indicated) of *EPR* fl/fl and *EPR* cKO mice and analyzed by qRT-PCR to detect *EPR* expression. The values are averages (±SEM) of three independent experiments performed in triplicate. Statistical significance (Student's t test) has been calculated using GraphPad Prism 9 for macOS and indicated. G. Left panel, macroscopic view of large intestine dissected from a representative

EPR fl/fl and a representative *EPR* cKO mice. Right panel, quantification of fecal pellets. The values are averages (±SEM) of eight independent mice per experimental group. Statistical significance (Student's t test) has been calculated using GraphPad Prism 9 for macOS and indicated.

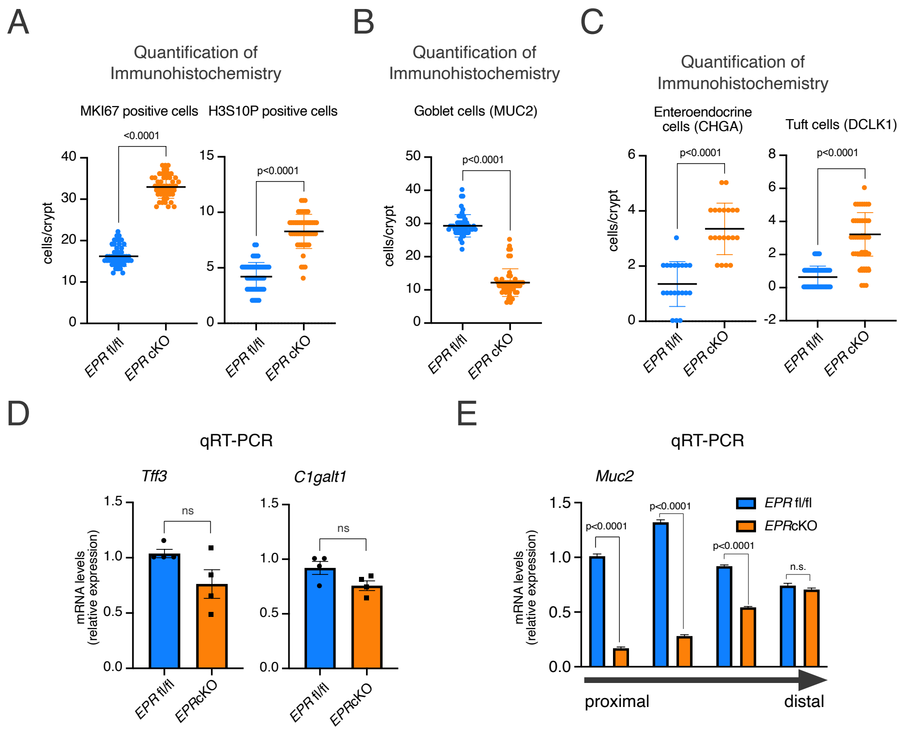

Supplementary Figure S2

Supplementary Figure S2.

A. - C. Non-overlapping images of immunohistochemistry experiments presented in Figure 1B were acquired and positive cells for each field were counted using the imaging analysis software package ImageJ 1.53a. Statistical significance (Student's t test) has been calculated using GraphPad Prism 9 for macOS and indicated. D. Total RNA was extracted from crypts purified from the upper colon of *EPR* cKO and *EPR* fl/fl mice (as indicated). E. Colon from either *EPR* fl/fl or *EPR* cKO mice were excised in 4 sections of equal length along the proximal-distal axis and the levels of *Muc2* quantified. qRT-PCR analysis was performed using transcript-specific primers. The values are averages (±SEM) of four independent experiments performed in triplicate. Statistical significance (Student's t test) has been calculated using GraphPad Prism 9 for macOS and indicated.

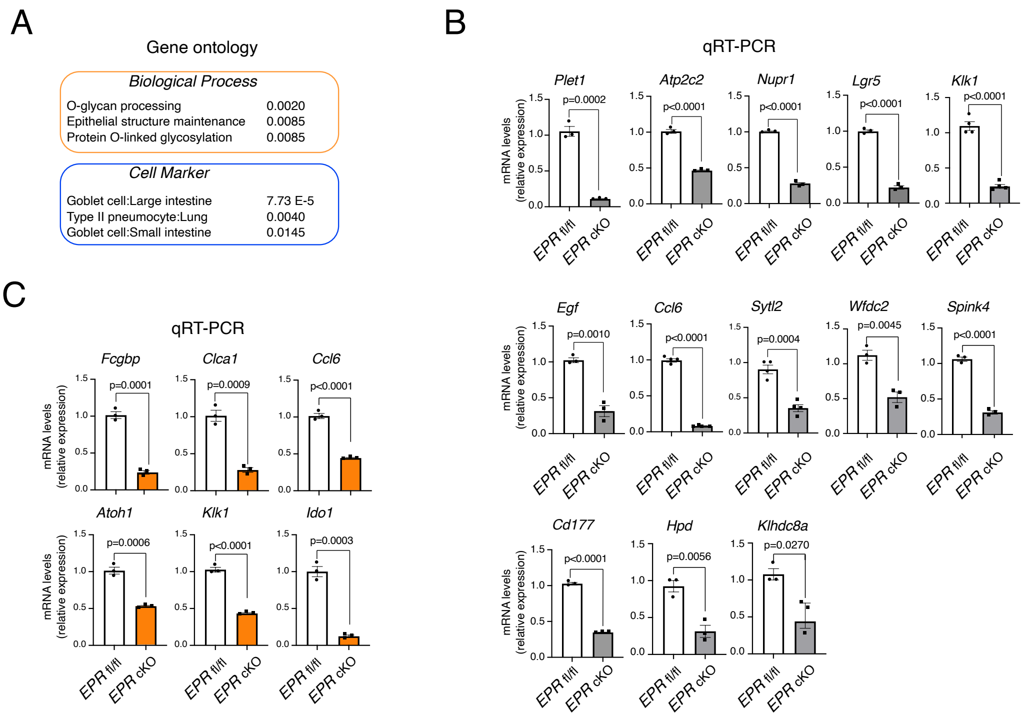

Supplementary Figure S3.

A. Gene Ontology analyses for Biological Process and Cell Marker (using the online EnrichR tool, https://maayanlab.cloud/Enrichr/) of transcript whose levels are down-regulated in large intestine of *EPR* cKO mice (see Figure 2). B. Total RNA was extracted from crypts purified from the upper colon of *EPR* cKO and *EPR* fl/fl mice (as indicated). qRT-PCR analysis was performed using transcript-specific primers. C. Total RNA was extracted from the distal third of ileum of *EPR* cKO and *EPR* fl/fl mice; qRT-PCR analysis was performed as indicated.

The mRNA level values are averages (±SEM) of three independent experiments performed in triplicate. Statistical significance (Student's t test) has been calculated using GraphPad Prism 9 for macOS and indicated.

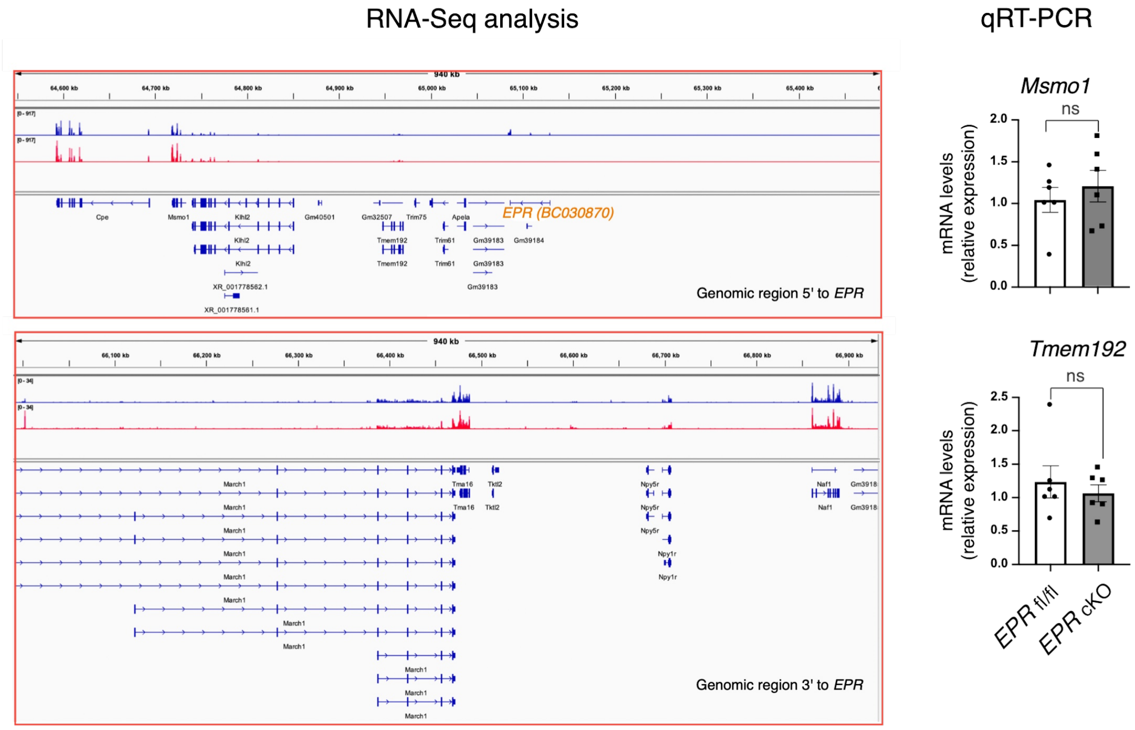

Supplementary Figure S4.

Left panel, snapshots of RNA-Seq results centered on the position of *EPR* (BC030870). Genes 5' to *EPR* are shown in the top panel while genes 3' to *EPR* are shown in the bottom panel as indicated). Genomic coordinates from Integrative Genome Viewer (Broad Institute) are displayed. Right panel, validation of the RNA-Seq results. qRT-PCR analysis of two neighbor genes of *EPR* performed on total RNA prepared from crypts purified from the upper colon of *EPR* fl/fl and *EPR* cKO mice.

The mRNA level values are averages (±SEM) of three independent experiments performed in triplicate. Statistical significance (Student's t test) has been calculated using GraphPad Prism 9 for macOS and indicated.

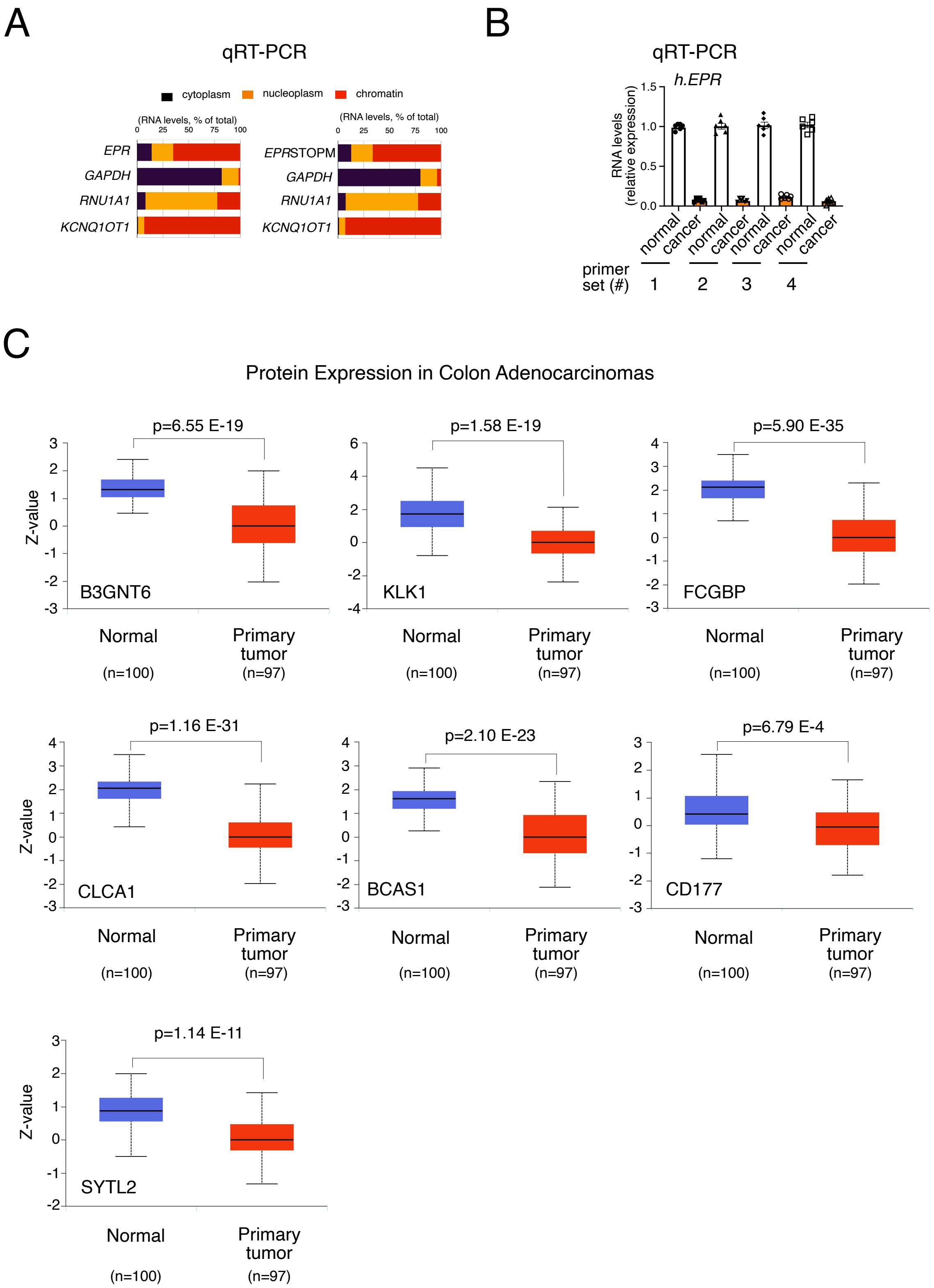

<u>Supplementary Figure S5</u>.

A. Either SW40-*EPR* (left) or SW480-*EPR*STOPM (right) were fractionated, RNA was prepared from cytoplasm, nucleoplasm, and chromatin, and analyzed by qRT-PCR to quantify

the indicated RNAs. B. *EPR* expression in colon adenocarcinomas and in the adjacent normal tissue has been quantified by qRT-PCR using four distinct sets of primer spanning different regions of the lncRNA (see Supplementary Table S2). Statistical significance (Student's t test for paired data) has been calculated using GraphPad Prism 9 for macOS and presented. C. Expression analysis of proteins corresponding to a set of *EPR* target genes in colon adenocarcinomas (and in the corresponding normal tissues) using the UALCAN tool which is designed to access publicly available datasets of Clinical Proteomic Tumor Analysis Consortium (CPTAC) Confirmatory/Discovery.

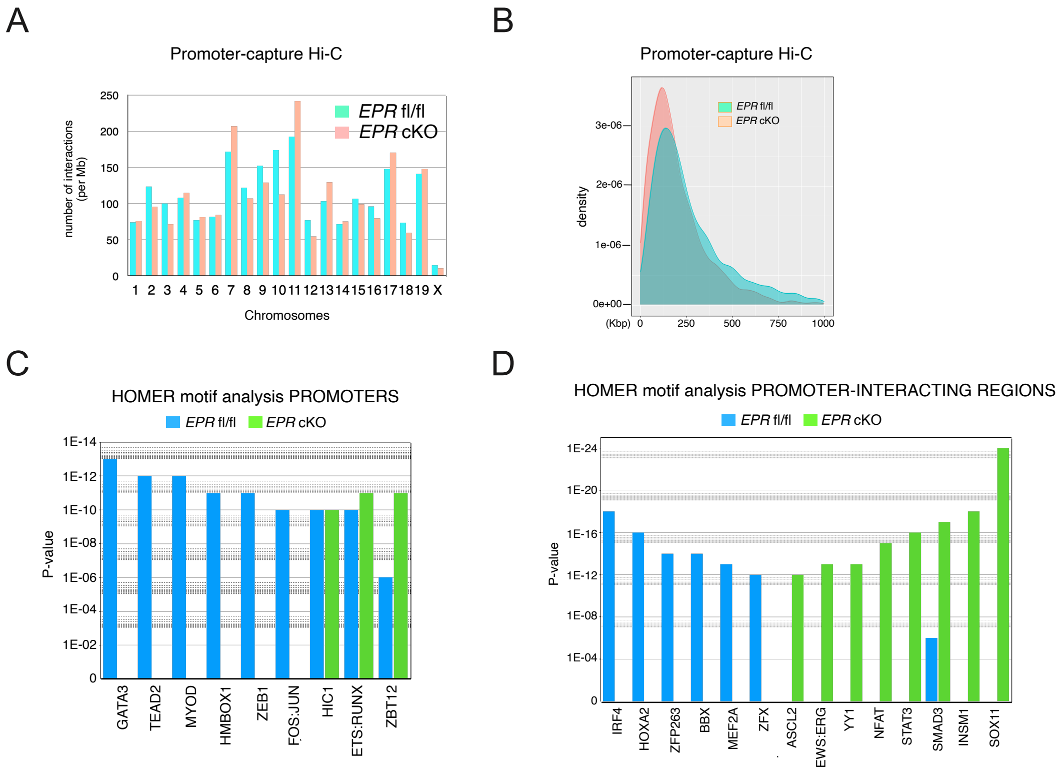

Supplementary Figure S6.

A. Number of Promoter-PIR intrachromosomal interactions (normalized per Mb). B. Density distribution of the distances spanning each Promoter-PIR interaction. C, D. HOMER de novo transcription factor binding motifs enriched in Promoters (panel C) and promoter-interacting regions (panel D) that are affected by EPR knock-out in promoter-capture Hi-C experiments. P-values for motif enrichment are shown.

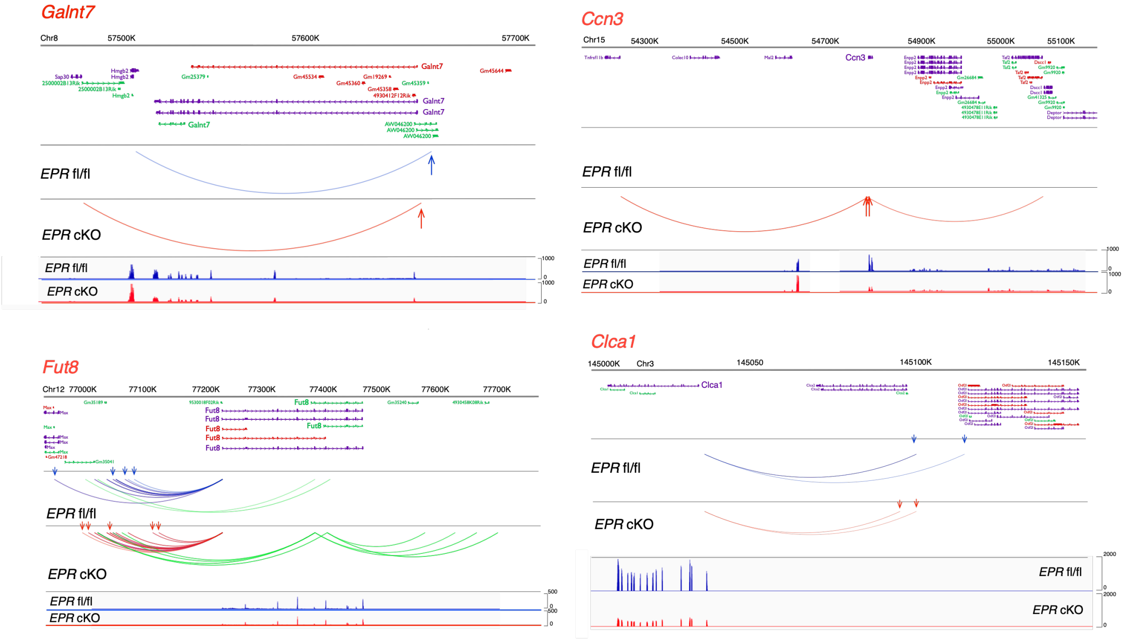

<u>Supplementary Figure S7</u>.

WashU EpiGenome Browser snapshot showing the interactions between the promoter region of the indicated genes and distal PIRs. Vertical arrows point to adjacent but distinct interactions. Green arcs regard *Fut8* alternatively spliced forms for which a CDS has not been defined. Snapshots of our RNA-Seq analysis are provided below (as indicated).

Supplementary Table

S1.

List of primary antibodies used for in this study.

ANTIBODY	SUPPLIER	CATALOGUE #
ACTB	Sigma	AC-74
CDH1 (immunoblot)	Santa Cruz	sc-31020
CDH1 (immunofluorescence)	Invitrogen	13-1800
TJP1 (ZO-1)	Invitrogen	33-9100
CLDN8	Sigma	AV33621
B3GNT6	Invitrogen	PA5-90159
CTNNB1	BD Transduction Laboratories	610154
MUC2 (immunoblot)	Invitrogen	PA5-10383
MUC2 (immunohistochemistry)	Santa Cruz	Sc-15334
ATOH1	Invitrogen	PA5-760-16
MKI67	Abcam	Ab16667
H3S10P	Cell Signaling Technology	9701
CHGA	Abcam	Ab15160
DCLK1	Cell Signaling Technology	62257
PLVAP	Abcam	Ab81719
CD8A	Abcam	Ab217344
CD4	Abcam	Ab183685
AIF1	Fujifilm	019-19741
H3K27me3 (CUT&RUN)	Cell Signaling Technology	9733
H3K27ac (CUT&RUN)	Abcam	Ab4729

Supplementary Table S2.

List of primers used for qRT-PCR, for constructing 3C-Seq libraries, and for 3C-PCR/qPCR analyses. The Illumina adapter sequences of primers used for 3C-Seq analysis are highlighted.

Primers for qRT-PCR	Forward	Reverse
mmu.Rpl32	CTGGCCATCAGAGTCACCAA	TGCACACAAGCCATCTACTCA
mmu.EPR	AACTGCGCAACCTAGTCCTG	GCTCCATGGAAGACTAACGG
mmu.B3gnt6	GCTGCACTGTAGCCACAAAC	TCAAGGGGACATCAGTGTGC
mmu.Atp2c2	GTGAGCGTCCTCACCAAAGA	TTCCAGAGACTTCTCCGACCT
mmu.Klk1	GCCCACTGATCTGTGATGGT	TCTGGTGTAGATACCCGGCA
mmu.St6gal1	GATGGTTTGGGACCGGTGT	GGACGCTTGGTCTCCGTTAT
mmu.A4gnt	CTTCCTGGGTACCAAGCACTAAT	CCTGAAGACAAGTGACCAGGAA
mmu.Galnt5	TGCTTGGTTCCGACGAGAG	TGCTTACCTGGAGTGGAGAGA
mmu.Plet1	GTCAGTGCCGTGATCCTGAA	ACTGCTGTCGTTGCACTTCT
mmu.Bcas1	TGCTCTGATAAAAACCCTCCCC	TTCACAGTCCAATTCAGTTCTCTGT
mmu.Nupr1	CGGAAAGGTCGGACCAAGAG	TCAGCAGCTTCCTCTCATGC
mmu.Sytl2	AACCTTGTGGCTCTTGCCTAT	CCTCAGACTAGCAACAATTCTCCT
mmu.Agr2	TGGACGATACTCAAACCGGC	TGCGCAGTTGGCTCTACAAT
mmu.Muc2	GAAGCCAGATCCCGAAACCA	GAATCGGTAGACATCGCCGT
mmu.Fgcbp	GGATGGAGGCAAGATCCGAG	ACAAGGTCATAGGCCACACG
mmu.Atoh1	GGGAAGCCCCGTGACAAATA	AAGGGGATTGGAAGAGCTGC
mmu.Spdef	GGAACCACCTACAGCACACA	CAGGAGCAGAGACCAGTGAC
mmu.Egf	AGGATCCTGACCCCGAACTT	ACTTCCGGTCTCGGCTTAAC
mmu.Clca1	AGCTGCAGGATGGAATCTTTGA	TCTATGGCGATGACGATGCC
mmu.Ccn3	GCCTATAGACCGGAAGCCAC	ACTCCGTCGTCTGCTCAATG
mmu.Cd177	GTGATCTGGCTCAGGACAGG	GAACTGTGGGTGTAGGTAGCTC
mmu.Ccl6	TTCGCCCTGCCACAATAGAG	ATTTCACCCCAAGAGCCCAG
mmu.Tff3	CCCTGGTGCTTCAAACCTCT	CAGATCGGGGATGCTTGCTA
mmu.Lgr5	CCTGGGAAAGCATACCCGTT	GGTTGACTCACAGGACCGTT
mmu.Hpd	AAGAGATGGGCGACCACTTG	CGCAGTCTTCCACCTCGAAT
mmu.Klhdc8a	CAGCAGCACAACAATTGGCA	GGAGAACACGGTGGGAGATG
mmu.Wfdc2	CTACTCAATCAGCGGGCCTT	CTCGGACACCTAAGCCTTCC

mmu.Fosb	CCAACCTGACGGCTTCTCTC	GAAGGGCTAACAACGGGGAA
mmu.Ido1	AGACAGCAATGGCACTCAGTAA	TGTGGTAGAGCAAAGCCCAC
mmu.Msmo1	AAAGGTTTCGGGAACTGGAGG	CAACTTCAGCCTCCTATGGCA
mmu.Tmem192	GTGCATGCTGGGAACCTGA	TTATAATCCACTCAGAGGCTCGC
mmu.Neat1	GGGACTTGTGGGAGAAAGCA	TTCCAGGCACAATCCTCACC
mmu.Rnu1a1	TGGCAGGGGAGATACCATGA	GTCGAGTTTCCCGCATTTGG
mmu.Fn1	GCAGGAAAGTCACCCAGACA	CTGTGGGAGGGGTGTTTGAA
mmu.Fstl1	ACGCTCCCACCTTCGCCTCT	GTCACCAGCGAGAGCGCCAG
mmu.Vim	TCCAGAGAGAGGAAGCCGAA	AAGGTCAAGACGTGCCAGAG
tRNA_lys	GCCCGGATAGCTCAGTCG	CCCGAACAGGGACTTGAAC
hsa.C1ORF43	GGATGAAAGCTCTGGATGCC	GCTTTGCGTACACCCTTGAA
hsa.EPR #1	TTTCATGGCCTGGTAGCGTT	GTCCCTGAGAGTGGCTAGGA
hsa.EPR #2	GGATGAGGCTCCATTCACTCC	GTGCCTCCCATTTCCAGTCA
hsa.EPR #3	CAGACTCACGGGTTCCTTCC	CTCCATGAACCACCACCGAA
hsa.EPR #4	TCATGGAGCTTCCCTACACCA	AGTGTAGATGGAAGCCAGGGT
hsa.FCGBP	CCTGGGGACGAGGACTTTTC	CACAGTAACCAGGAGGCGTT
hsa.SYTL2	TCGGAGGTAGGTGATGCTCT	TCGCTTGTTCCTATGGTGGC
hsa.B3GNT6	CAAGAAGTCAGCCAACACGC	CTGGTTTCCACTTTGCACGG
hsa.CLCA1	TGCAAGCAAGCTCACAAACC	TGCTGGTGTCCTTGTTCGTT
hsa.BCAS1	AGAAGACACGGGTGCTGAAAA	CCTTTGGAATTCTTGCCAGCC
hsa.CD177	TCCTGCTAACTCTATTACCCCCA	ATGAGGTCAGAGGGAGGTTGA
hsa.ATOH1	CAGCTGCGCAATGTTATCCC	TTGTAGCAGCTCGGACAAGG
hsa.KLK1	TGTCACCATGTGGTTCCTGG	ACAATCCGGGACTGAATCGG
hsa.CASP9	GGACAGATGAATGCCGTGGA	CACAGGGATCATGGGACACAA
hsa.GADD45B	TACCGTTGGTTTCCGCAACT	CTTCCCACAGAGCTAGAGCG
hsa.DDIT3	CCGAGCTCTGATTGACCGAA	GGGAAAGGTGGGTAGTGTGG
hsa.RASSF2	ACTTAAATGGCAGGGGGTGG	CAGCCTTGTGCACGTTCTTC
hsa.NGFR	ACATTCCGACAACCGATGCT	CAGAGGAGCTCAGTTCTGCC
Primers for Genotyping	Forward	Reverse
LoxP	TGAGATGACTGCACCAAGAC	ACCTCTCCACTCCTTGAAAC
CRE	AGGAGCCAGCGGAGCAC	ACATGTCCATCAGGTTCTTGC
Primers for 3C-qPCR analysis	Forward	Reverse

Bait_1	TATTCTTGCATTTGACCAATCCAGG	
Bait_2	GCCTTCATCTACTCTCCTTCTCATC	
Bait_3	CACTGATCTCACCTGCCTTTATTAC	
PIR_1		CATAGGACATGCTGGGACCC
PIR_2.1		GTAGAGCCACCATTCACTTAAAGAT
PIR_2.2		AATCACCACCCATCAGCCCCTTTTA
PIR_2.3		TCAGACCCAAAATGACTAAACATCA
Primers for CUT&RUN-qPCR analysis	Forward	Reverse
C&R.*B3gnt6*	GGTGCTGAATCAGTGGTTCCT	GCCAAAGACCGGCGAGT
C&R.*Galnt5*	CCAGCCTAGTCCCACGTTTT	GCATCTTGTCTCACAGGGCT
C&R.*St6gal1*	ACTGCCCGACCCTAACAAAG	GCAGCTCTCTCAATCGGGAA
C&R.*Bcas1*	ACCCAAGATACTCCCACCGA	AGCAATAGACACTCCAGCGG
C&R.*Rpl30* Intron2	Cell Signaling primer set #7015	Cell Signaling primer set #7015
Primers for ChIRP-qPCR analysis	Forward	Reverse
ChIRP.*B3gnt6*	GGTGCTGAATCAGTGGTTCCT	GCCAAAGACCGGCGAGT
ChIRP.*St6gal1*	ACTGCCCGACCCTAACAAAG	GCAGCTCTCTCAATCGGGAA
ChIRP.*Galnt5*	CCAGCCTAGTCCCACGTTTT	GCATCTTGTCTCACAGGGCT
ChIRP.*Rpl32*	ACATTTGCTCAACCAACCGC	GCCCTAAGTGGAAGCCCAAT
